# What do we learn when we adapt to reading regional constructions?

**DOI:** 10.1371/journal.pone.0282850

**Published:** 2023-04-07

**Authors:** Julie E. Boland, Emily Atkinson, Guadalupe De Los Santos, Robin Queen

**Affiliations:** University of Michigan, Ann Arbor, MI, United States of America; University of Birmingham, UNITED KINGDOM

## Abstract

We present four experiments investigating adaptation to a regional grammatical structure through reading exposure, using both the *needs* + past participle construction (e.g., *The car needs washed*) and the double modal construction (e.g. *You might could go there*). In each experiment, participants read two stories containing informal dialogue. Half of the participants were exposed to one of the regional constructions and half were not. Those readers exposed to the regional constructions adapted, gradually reading the novel constructions faster over 9 to 15 exemplars. The degree to which the exposed group learned the construction was tested in two ways. In the first two experiments, learning was measured by comparing reading times to acceptable and unacceptable variants of the novel constructions. Readers did not learn either the verb tense rule for the needs construction (Experiment 1) or a simple ordering rule for double modal constructions (Experiment 2). Similarly, in Experiments 3 and 4, metalinguistic judgments used to test learning revealed that participants had failed to acquire the regional grammar of either novel construction. These experiments suggest that the adaptation effects reflect learning some general properties of the experimental stimuli, not learning the syntactic constructions themselves.

## 1. Introduction

As children, we learn a local variety of our native language. All languages vary in pronunciation, lexicon, and grammar, depending on the country, region, and ethnic community in which one learns them. Throughout our lives, most of us also have some exposure to other varieties of our native language, whether through travel, interaction with people from other regions/ethnicities, or watching television and film. While this exposure may not change the way we speak, it could expand our linguistic knowledge to encompass other exposed varieties to some degree. For example, through exposure to southern varieties of English, a northerner may learn to better recognize and comprehend those varieties, even without adopting a southern way of speaking. Perhaps, such a northerner would also learn the grammar of certain southern constructions, and be able to make Southern English grammaticality judgments. Certainly, we would expect such grammatical competence after a northerner has lived in the south for many years. Thus, our American English grammar might include both our own local variety and (at least some constructions from) other varieties.

In this article, we investigate adult learning of two regional syntactic constructions through brief written exposure, similar to reading a short story in which the characters speak in dialect. We are especially interested in what participants learn about the grammar of these constructions. One of the constructions has previously been studied in adaptation paradigms and the other has not.

### 1.1 Adaptation to novel constructions

In a groundbreaking paper, Kaschak and Glenberg found that readers quickly adapted to a novel regional construction known as the *needs* construction [1] (e.g., The dishes need *washed*, which means the same thing as the Standard American English (SAE) form, *The dishes need to be washed*). When readers first encountered the novel construction, reading times slowed dramatically a few words after the construction, indicating comprehension difficulty. However, within just ten exposures, reading times were no longer elevated, and furthermore, readers seemed to be able to generalize whatever they had learned to other variants of the construction [1, 2]. Most experiments also included evidence of generalization in the second half of the experiment to new verbs (e.g., *The cat wants petted*) and/or new syntactic formulations (e.g. *John thinks that what the meal needs is cooked*). Kaschak interprets this generalization as evidence of “abstract knowledge about the structure of the construction, rather than simply learning a rule about its surface structure” [2].

Similarly, other researchers, using a diverse array of experimental paradigms, have found that readers, listeners, and speakers rapidly adapt to the experimental stimuli of a particular study [3–6]. For example, in a visual world eye-tracking study, Kamide found that listeners learned the relative clause attachment patterns of several different speakers [4]. In an ERP experiment, Hanulikova et al. found that by the second half of an experiment that contained many gender agreement errors, Dutch listeners no longer had a typical P600 response to the errors, even when produced by native speakers [5]. Likewise, Luka and Choi found that reading aloud tokens of an unusual syntactic structure improved grammaticality judgments of other sentences using that same structure days later [6]. These and other studies suggest that grammatical performance is malleable, rapidly adapting to recent input.

### 1.2 Does adaptation entail learning?

Is grammatical competence also influenced by recent out-of-dialect input? While adaptation was rapid in the above studies, passive reading of isolated sentences is not an ideal situation for implicit learning of grammar in adult second language acquisition [7]. For example, Wang et al. found that production of a new syntactic structure was more likely when text exposures to that structure were enhanced by illustrations or video [8]. Relatedly, children learn their first language via live interaction, not passive listening. Classic studies on language socialization and its effect on first language acquisition reveal that, even if a community does not believe in direct adult-child communication, infants are still receiving input from and interaction with other community members, e.g., their siblings, other children, lower status caregivers [9, 10].

In the current paper, we use a version of Kaschak’s [2] self-paced, word by word reading time paradigm to further investigate learning of the *needs* construction (Experiments 1 and 3) and to begin investigation of the double modal construction (Experiments 2 and 4). We focus on the issue of what has been learned during adaptation, and whether exposure leads to grammaticality judgments that match the intuitions of native speakers of the dialect. Previous reading time studies of grammatical adaptation have required participants to read lists of isolated sentences [1–3, 11]. We extended prior research in several ways. First, we embedded the regional constructions in a naturalistic story context, designed to mimic ordinary exposure. Second, we tested learning in the second phase of the experiment, either by comparing reading times for acceptable vs. unacceptable forms of the regional construction (Experiments 1 and 2), or including a survey of metalinguistic judgments (Experiments 3 and 4). Our primary goal was to better understand the type of learning reflected by adaptation effects during reading exposure.

### 1.3 The constructions

Before presenting the experiments, we briefly review the two constructions, to clarify what grammatical features must be learned in order to make metalinguistic judgments about them. The *needs* construction consists of a subject noun phrase followed by *need(s)*, followed by a past participle verb. Its usage is somewhat narrow: Murray et al. [12] reported that only half of users accept the negative form (e.g., *The car doesn’t need washed*) and that the construction may be restricted to particular chores (e.g., washing dishes and changing diapers). The double modal construction is named for the two modal verbs that occur sequentially (e.g., *I might could do that*), something that does not occur in SAE. The double modal construction is found mostly in Southern US English. It tends to occur during negotiation contexts, along with hedges and politeness expressions [13, 14]. For example, Mishoe and Montgomery point out hedges in this example from a negotiation context “Do you think we can afford it, or should we might cancel the trip?” [13]. And in an experiment, Hasty found that residents of northeast Tennesee (aged 21–75 years) judged a doctor as sounding more polite when telling a patient “We may can add the Pulmicort, which is a steroid” compared with “We may add the Pulmicort, which is a steroid” [14].

The *needs* construction and the double modal construction both represent syntactically unusual verb phrases that are strongly lexically restricted: some users of the *needs* construction use it only with the verb *needs*, while others also use the construction with *want* [12]. The double modal construction occurs most often with *might* or *may* as the first modal, and the order of the modals is fairly rigid [13]. Both constructions are interpreted as ungrammatical (often strongly) outside of their general region of use. Furthermore, neither construction is socially stratified within its primary region of use.

## 2. Experiment 1

The goals of this experiment were (1) to expose participants to the *needs* construction in naturalistic dialogue, in order to create an optimal context for learning it and (2) to test participants’ knowledge of the *needs* construction in a similar naturalistic context, using the implicit measure of reading time. In addition, we expected to replicate earlier work demonstrating that readers initially have slow reading times for the *needs* construction, but adapt (i.e., gradually decreasing reading times) with increasing exposure [1–3, 11].

Claims about the acquisition of the *needs* construction made by Kaschak and colleagues rest on changes in reading time that occurred across each experiment, and the contrast in reading times between participants trained on the *needs* construction and participants exposed to the standard *needs to be* construction [1, 2]. The data patterns reported in the Kaschak studies would be expected if participants were learning and generalizing a new syntactic form. However, the interpretation of these data is clouded by the lack of a control condition in which participants are asked to read ungrammatical generalization sentences. This is a critical omission from the earlier experiments, as it has been shown that participants exposed to grammatical errors throughout an experiment show an attenuated response to those errors by the end of the experiment [5]. Without an ungrammatical control condition, we cannot distinguish the claim that participants are generalizing the *needs* construction to new syntactic forms (e.g., after training on *The meal needs cooked*, participants readily process, *John thinks that what the meal needs is cooked*) from the claim that exposure to the *needs* construction blunts participants’ sensitivity to grammatical errors within the experiment.

We addressed this shortcoming as follows. Following Kaschak’s approach [1, 2], we used a self-paced, word-by-word sentence reading paradigm. The first part of the experiment exposed participants to the *needs* construction in a between participants design. One group of participants read a story that contained ten tokens of the *needs* construction, and another group read the same story, in which the standard *needs to be* construction was substituted. Then participants read a second story designed to assess their grammatical knowledge of the *needs* construction. Half of the participants in each exposure group saw acceptable needs constructions in the second story (e.g., *It needs scrubbed*) and half of the participants saw ill-formed, unacceptable instances (e.g., *It needs scrub*). Participants in the Control group should read both types equally slowly. However, participants who were exposed to the *needs* construction during the first story, should behave differently from the Control group. If the exposed participants have learned the grammatical restrictions of the construction (i.e., that the verb following *needs* must be a past participle), then they should read acceptable *needs* constructions faster than the unacceptable ones. However, if exposed participants have simply learned to expect constructions outside of their grammar, then the Exposed group should read both acceptable and unacceptable types faster than the Control group. This latter result would undermine claims that participants were truly generalizing the construction to new forms in prior studies.

### 2.1 Methods

#### 2.1.1 Participants

We recruited 90 undergraduates from the Psychology Subject Pool. Participants received partial credit in an introductory Psychology class for their participation. This study was reviewed and determined exempt by the University of Michigan IRB.

#### 2.1.2 Materials

Participants read a story about a woman preparing to go out of town, who first had a conversation with her house sitter (Phase 1) and then had a conversation with her traveling companion (Phase 2). The stories are included in S1 Appendix and an example of a critical sentence from each story is provided below in (1) and (2). Both conversations were focused on household maintenance and chores, a domain in which users of the *needs* construction report using it. In contrast with prior experiments, all occurrences of the *needs* construction occurred within quotations, and other uses of *need* and *needs* were included (although there were relatively few). The conversations included other types of colloquial language, such as informal contractions (e.g., *wanna*) and informal idioms (e.g., to *wolf down* food). Words and phrases implying fast or slow speech were avoided; prior research has shown that quoted dialogue is read faster if it is implied that the speaker is talking quickly [15]. There were two versions of each story. During Phase 1, there were 10 tokens of the *needs* construction (training condition) or the semantically equivalent *needs to be* constructions (control condition). During Phase 2, there were five acceptable or unacceptable tokens of the *needs* construction.

(1) *I’ve already fed the dog today*, *but he will need fed (need to be fed) every morning around 8*:*00*. Phase 1, Needs version (Control version)
(2) *It would need scrubbed (need scrub) top to bottom and it’s all very dicey*. Phase 2, Acceptable version (Unacceptable version)

#### 2.1.3 Procedure

Participants were tested in the lab. After reading and signing an informed consent form, participants were randomly assigned to one of four participant groups, representing one of the cells in our 2 (exposure during Phase 1) by 2 (regional acceptability during Phase 2) design. Participants read two stories containing mostly informal dialogue. For half of the participants, the first story (Phase 1) contained ten *needs* constructions (Needs group). The other half of the participants (Control group) read Standard English versions (e.g., *needs to be…*). In the second story (Phase 2), half of the participants from each of the Needs and Control groups read five unacceptable, and presumably ungrammatical, constructions using *needs* (e.g., *needs wash*). The other half read five regionally acceptable *needs* constructions (e.g., *needs washed*).

The sentences in the stories were presented in the moving window style, with reading times collected for each word [16]. Multiple short sentences were sometimes presented together on a single line. For each critical sentence, the first button press brought up a left-aligned fixation point, followed by a row of underscores on the same horizontal row. Each underscore represented a letter in the sentence, with no underscore for spaces between sentences. Upon each button press, a word appeared, moving from left to right across the screen. At semi-regular intervals, two comprehension questions appeared, one at a time, on the screen. Participants answered the question by pressing a button labeled "yes" or a button labeled "no." Prior to the stories, participants were given four practice trials, in order to become familiar with the procedure.

After reading the two stories, participants were asked two questions about their familiarity with the needs construction: how often had they heard it (never; only once or twice; regularly, but not often; frequently; very often), and in how many different contexts they had experienced it (never heard it; only in books/movies/TV; used by someone they do not really know; one or more of their friends uses it; one or more of my relatives uses it; use it themselves). This measure provides a rough index of prior exposure to the construction in our sample and the responses to these questions for all four experiments can be found in S4 Appendix. The responses may overestimate prior exposure due to recent exposure during the experiment, particularly for the groups exposed to the construction during Phase 1, so it is not used in the analyses. Participants also completed a 44-item Big Five Personality questionnaire [17], which is not reported here. The entire experiment took about 25 minutes.

#### 2.1.4 Analysis

The final stimulus item from the ten Phase 1 items was not analyzed, due to a stimulus error. Participants whose accuracy on the comprehension questions was less than two standard deviations below the mean (i.e., accuracy less than 73%) were dropped from the analysis. Four participants were dropped for this reason–one from each of the four participant groups. The critical regions were identical for the exposure (Phase 1) and test (Phase 2) blocks: *need(s)*, the critical verb, and the spillover region, which consisted of the word following the critical verb. The critical regions are illustrated with an example in Table 1, using the example item from (1). Following Kaschak’s methods [1, 2], we did not analyze or graph reading times for the words “to and “be,” which always followed “need(s)” in the Control condition of Phase 1. This means that the verb (*fed*) was preceded by different words in the two conditions, and occurred two words later in the Control condition, compromising the interpretation of Exposure condition at the verb region. However, based on Kaschak’s prior work, we expected to see the effects of Exposure condition at the verb+1 region, where reading times in both conditions should be heavily influenced by the previous word (*fed*). The same issue does not arise during Phase 2, when the “to be” form is not used in our stimuli.

**Table 1:** Critical regions (in gray) for analysis and figs, for Experiments 1 and 3.

	need(s)–1	need(s)	verb	verb+1	verb+2	verb+3
**Needs Condition**	will	need	fed	every	morning	around
**Control condition**	will	need (“to” & “be” ignored)	fed	every	morning	around

Outliers that were greater than three standard deviations from the mean of the individual regions were removed. In the exposure block, this resulted in a total data exclusion of 2.1% (0.6% from the *need(s)* region, 2.7% from the verb region, and 3.0% from the spillover region). In the test block, a total of 2.2% of the data were excluded (1.2% from the *need(s)* region, 3.1% from the verb region, and 2.6% from the spillover region).

Reading times were analyzed using linear mixed effect models with exposure condition, item order (1–9 in Phase 1, 1–5 in Phase 2), and their interaction as fixed effects, and participants as random effects. For each model, the maximal random effect structure that converged was utilized [18]. For the Phase 2 analysis, regional acceptability and its interactions were also included as fixed effects. Exposure condition and regional acceptability were Helmert-coded (*Needs group* = 1, control group = -1; acceptable = 1, unacceptable = -1). Identical models were used to analyze reading times at each word in the three-word critical region. For all four experiments, statistical models were run in the R environment [19] using the lme4 package [20]. Estimates for *p*-values for the fixed and random effects were calculated using the Sattherwaite approximation in the lmerTest package [21].

### 2.2 Results

#### 2.2.1 Phase 1: Exposure block

**Fig 1:**
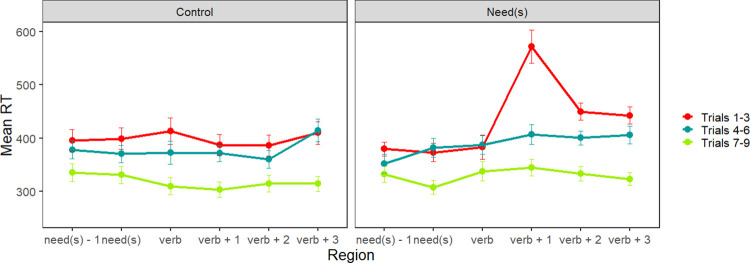
Experiment 1 exposure block. Mean reading times for critical sentence regions by trial number grouped by early (trials 1–3), middle (trials 4–6) and late (trials 7–9). Data from the Control group are presented on the left, while data from the Needs group are presented on the right.

Reading times for the first story (i.e., the exposure block) for both exposure groups are plotted in Fig 1. In order to illustrate adaptation over time, the critical sentences were grouped into three time domains (although the statistical analysis was run with raw, ungrouped order as a factor): early trials (1–3), middle trials (4–6), and late trials (7–9). Both groups’ reading times in the critical regions decrease with additional trials, but it is clear that the *needs* exposure group has particularly slow reading times in the early trials, i.e., upon their first exposures to the *need(s)* + past participle construction.

The analysis of the exposure phase is summarized in Table 2. In the models for the *needs* and verb region, the maximal random effect structure that converged contained a random intercept and a random slope for order; in the spillover region, only a random intercept was used. In both the *needs* region and the critical verb region, there was an effect of trial order; participant’s reading times decreased with each additional trial.

**Table 2:** Experiment 1 exposure block fixed effects summary for the three regions of analysis: The *need*(s) region, the critical verb region, and the spillover region (i.e., verb + 1).

	*Need(s)* Region		Verb Region		Spillover Region	
	*β*	*SE*	*β*	*SE*	*β*	*SE*
Exposure group	-7.20	144	-20.27	14.52	103.16 ***	15.52
Trial order	-11.55 ***	1.85	-11.97 ***	2.01	-25.64 ***	2.00
Exposure x Order	0.35	1.85	3.84 †	2.01	-11.95 ***	2.00

Note:

† p < 0.1

* p ≤ 0.05

** p ≤ 0.01

*** p ≤ 0.001

Crucially, in the spillover region (i.e., verb + 1), there was an effect of exposure group and trial order. Both groups adapted across the task, but the Needs group had overall slower reading times in this region than the Control group. Crucially, there was an interaction of exposure group and order, such that participants in the Needs exposure group adapted more strongly than those in the Control group, as expected.

#### 2.2.2 Phase 2: Test block

**Fig 2:**
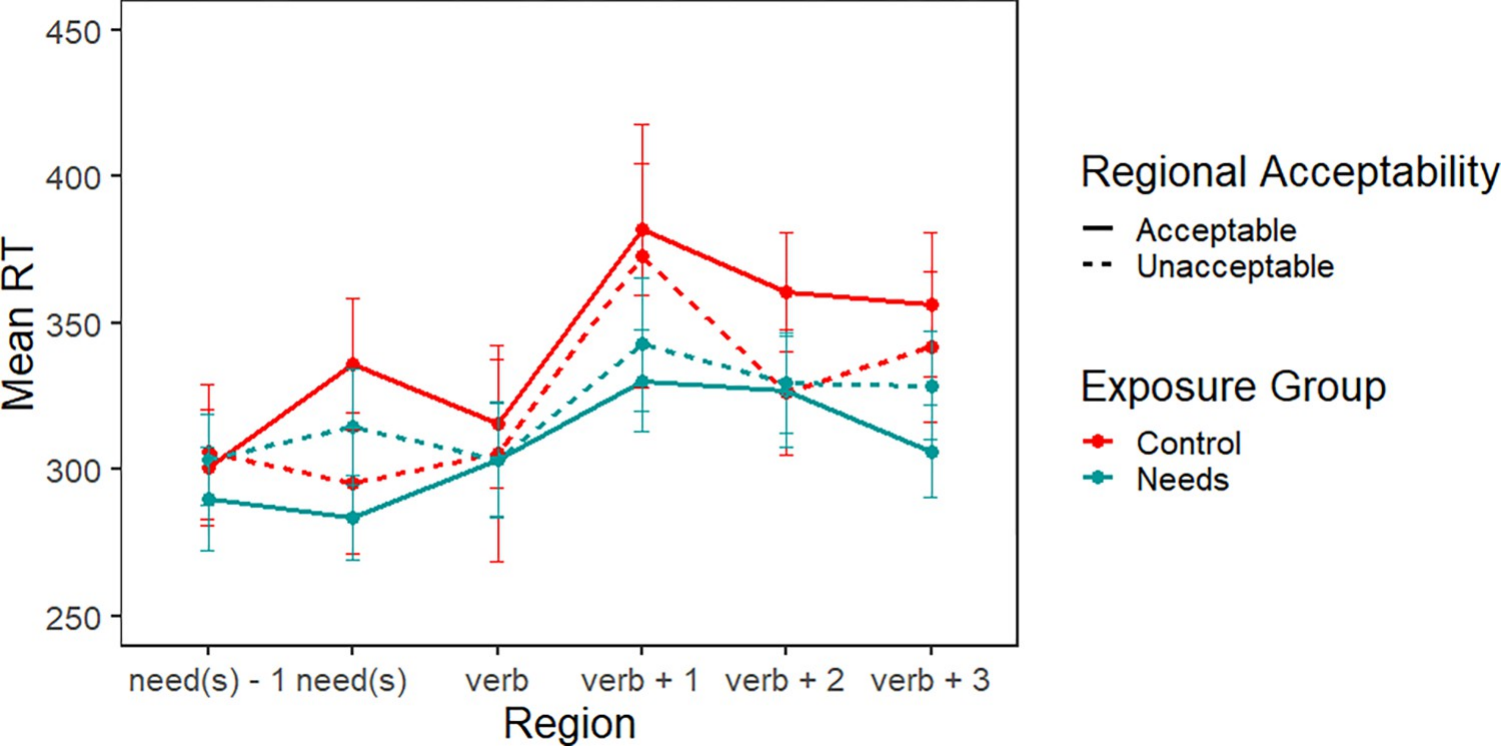
Experiment 1 test block. Mean reading times for critical sentence regions by acceptability of the regional structure (acceptable vs. unacceptable) and exposure group (Control vs. Needs).

Fig 2 presents the reading times for the second story (i.e., the test block) for both exposure groups and types of regional sentences. We included only five critical sentences, to minimize potential exposure-based adaptation effects, especially in the Control group. Reading times were overall faster in Phase 2, compared with Phase 1, without large differences among conditions. In contrast to Fig 1, the vertical access is zoomed in, and each point represents data averaged over five trials rather than three. If participants learned the *needs* construction through exposure in Phase 1, we should see an interaction of exposure group and regional acceptability, with only the Needs group exhibiting faster reading times to the regionally acceptable *needs* construction.

**Fig 3:**
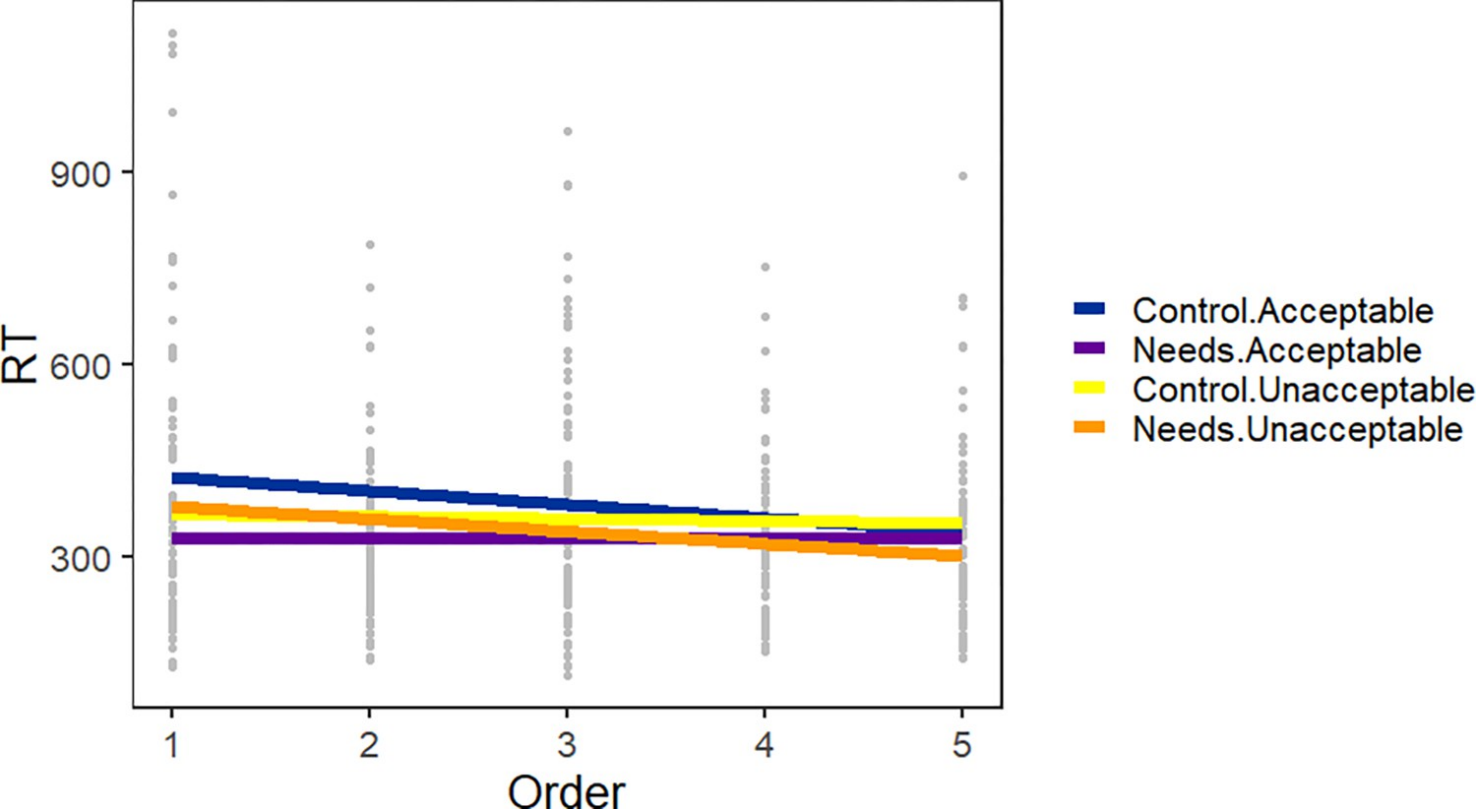
Experiment 1 test block reading times in the spillover (verb + 1) region. Plotted by the order of the five test trials, and the intersection of exposure group (control vs. *need(s)*) and regional acceptability (acceptable vs. unacceptable).

Table 3 summarizes the statistical analysis of the test phase of Experiment 1; the random effects structure was identical to that from the exposure phase–random intercepts in all three regions and random slopes for order in the *needs* and verb regions. In both the *need(s)* and critical verb regions, there were no significant effects. In the spillover region, there was an effect of trial order indicating that participants continued to adapt to the task (i.e., read progressively faster) during this second phase of the experiment. There was a marginal interaction of exposure group and regional acceptability (*p* = 0.07)–i.e., the interaction predicted by the learning hypothesis. However, there was no difference between the regionally acceptable and unacceptable version for either group in planned pairwise comparisons (all ps > 0.1). Finally, there was a 3-way interaction between exposure group, regional acceptability, and trial order. This interaction (see Fig 3) reflects the fact that the Control + Acceptable group read progressively faster, while the Control + Unacceptable group’s reading times did not change over the five trials. Conversely, the Needs + Acceptable group’s reading times did not change, but the Needs + Unacceptable group read progressively faster.

**Table 3:** Experiment 1 test block fixed effects summary for the three regions of analysis: The need(s) region, the critical verb region, and the spillover region (i.e., verb + 1).

	*Need(s)* Region		Verb Region		Spillover Region	
Predictor	*Β*	*SE*	*Β*	*SE*	*Β*	*SE*
Exposure group	1.16	16.53	5.45	18.96	-22.14	19.33
Regional acceptability	14.54	16.53	1.50	18.96	0.68	19.33
Trial order	1.65	3.82	-5.33	3.68	-10.69 *	4.78
Exposure x Acceptability	-26.90	16.53	0.55	18.96	-35.04 †	19.33
Exposure x Order	-3.21	3.82	-1.94	3.68	0.84	4.67
Acceptability x Order	-3.87	3.82	1.11	3.68	-0.10	4.67
Exposure x Acceptability x Order	2.92	3.82	-1.84	3.68	9.60 *	4.67

Note:

† p < 0.1

* p ≤ 0.05

** p ≤ 0.01

*** p ≤ 0.001

#### 2.2.3 Bayes analysis

These results do not demonstrate that participants who adapted to the *needs* construction during Phase 1 learned the *need(s)* + past participle pattern. Even so, traditional hypothesis testing can only tell us that there is no evidence against the null hypothesis, not provide evidence *for* the null. Bayes factors (BFs), i.e., the ratio of the likelihood of the alternate hypothesis to the likelihood of the null, can provide us this stronger evidence for (or against) the null. BFs can range from less than 1/100 to greater than 100. A BF of 1 indicates no evidence for either hypothesis because the likelihoods are the same. A BF less than 1 indicates evidence for the null, while a BF greater than 1 indicates evidence for the alternative hypothesis. We conducted three such analyses, both focusing on the Phase 2 spillover (verb + 1) region, and using the Bayes Factor package [22] set to 100,000 iterations.

First, we examined the hypothesis that exposure to the *needs* construction affects performance in Phase 2 in general, i.e., that the Needs group is distinguishable from the Control group in the spillover region. To do this, we calculated a Bayes factor comparing the full model to a model without the exposure group factor and its interactions. The BF of 0.26 ±2.79% indicates moderate evidence [23] for the simplified model.

Despite the lack of evidence that the exposure group impacted reading times in Phase 2, we also tested our primary hypothesis that learning the construction should lead to an exposure by acceptability interaction. A Bayes factor comparing the full model to the model minus this interaction was calculated. Evidence for the null would indicate that the Needs group does not respond to variations in regional acceptability differently than the Control group. The Bayes factor of 1.00 ±2.79% for the spillover region indicates anecdotal evidence for the learning hypothesis, but note how close this value is to equal likelihood for both models (i.e., a BF of 1, which is indicative of no evidence in either direction).

Finally, to further corroborate this finding, we calculated a Bayes factor for each individual exposure group comparing a full model with the grammaticality of the sentences interacting with order to a null model without the grammaticality factor and its interactions. For both groups, the Bayes factor for the spillover region indicates anecdotal evidence for the null hypothesis (Needs: 0.29 ±1.16%, Control: 0.29 ±1.47%). Thus, neither group distinguished the regionally acceptable sentences from their ungrammatical counterparts.

### 2.3 Discussion

The informal, conversational prose employed as stimuli for this experiment worked well in producing adaptation effects during Phase 1. Exposure effects were localized in the spillover (verb+1) region, consistent with several prior experiments [1, 2]. The adaptation effects are likely to be composed of at least two mechanisms: cumulative priming and learning. These two phenomena are closely related; in fact, many syntactic priming effects are characterized as implicit learning [24–26]. In self-paced reading time paradigms, adaptation and priming are both measured by reductions in reading times and are therefore indistinguishable. However, we use the term “adaptation” rather than priming because priming is generally understood to be limited to known words and known syntactic constructions. Because the constructions investigated here may be completely novel for some participants, the observed adaptation effects likely involve some combination of learning (i.e., developing mental representation for the constructions and procedures for processing them) and cumulative priming (further refining and strengthening the nascent representations and procedures). In addition to construction learning, participants might also be learning experiment-specific contingencies and tuning expectations about upcoming words and phrases in the experiment. We use the term “adaptation” to include all of these cognitive phenomena.

Despite adaptation to the *needs* construction during Phase 1, there was no clear evidence that the Needs group learned that the verb following *needs* is in the past participle tense (i.e., an–*ed* ending for most verbs). Although a marginal exposure by acceptability interaction was observed during Phase 2, the learning hypothesis predicted faster reading times by the Needs group for regionally acceptable *needs* sentences, compared to unacceptable *needs*, and no such difference was found. To be clear, the data pattern for the Needs group showed numerical differences in the predicted direction (Fig 3, spillover region). Further, the targeted Bayes factor analysis showed approximately equal weight for the learning hypothesis and the null hypothesis. One word later (outside of our critical region), the observed data pattern was inconsistent with the predicted learning effect because the Needs group read both acceptable and unacceptable forms as quickly as the Control group read the unacceptable form. Most definitively, a Bayes analysis testing whether exposure to the *needs* construction during Phase 1 impacted reading times more generally in Phase 2 showed moderate support for the null hypothesis, i.e. exposure to the *needs* construction during Phase 1 had no effect on reading times during the Phase 2 spillover region.

## 3. Experiment 2

This experiment extends prior adaptation effects to a new regional construction. The goals were (1) to determine whether readers adapt to double modal constructions just as they adapted to *needs* constructions in Experiment 1, using similar naturalistic stories as the context for learning, and (2) to measure learning of the double modal construction by contrasting acceptable and unacceptable forms during the second story, analogous to Experiment 1.

### 3.1 Method

#### 3.1.1 Participants

One hundred thirty-seven college students were recruited from the Psychology Subject Pool.

#### 3.1.2 Materials & procedure

Participants were tested in the lab. After reading and signing an informed consent form, participants were randomly assigned to one of four participant groups, representing one of the cells in our 2(exposure during Phase 1) by 2(acceptability during Phase 2) design. Participants read two stories containing mostly informal dialogue (see S2 Appendix). For half of the participants, the first story (Phase 1) contained ten double modals for which the first modal was always *might* or *may* (DM condition). The other half of the participants (Control condition) read SAE approximations (e.g., *You probably should…*). In the Control condition, either the first or second modal was replaced with a high frequency adverb (e.g., *probably*, *just*). In the second story (Phase 2), half of the participants from each of the DM and Control conditions read five unacceptable reversed double modals (i.e., the second modal verb was *might* or *may*). The other half read five acceptable double modals beginning with *might* or *may*. Example critical sentences are provided below in (3) and (4).

(3) *I was thinkin’ you might could (might just) look at it quick*, *since it was just here*. Phase 1, Double Modal condition (Control condition)
(4) *Come on in*, *you might can (can might) help us pack*. Phase 2, Acceptable condition (Unacceptable condition)

Stimuli were presented in the same moving window style as in Experiment 1, with the same button-pressing task and analogous comprehension questions. After reading the two stories, a subset of participants answered questions about how often they had heard, and their familiarity with, the double modal construction, which used the same scales as Experiment 1. All participants completed a 44-item Big Five Personality questionnaire, which is not reported here. The entire experiment took about 25 minutes.

#### 3.1.3 Analysis

The analysis for the double modal experiment was similar to that for the *needs* construction experiment (Experiment 1). Participants whose accuracy on the comprehension questions was less than two standard deviations below the mean (i.e., accuracy less than 78%) were dropped from the analysis. This resulted in eight participants being dropped–six from the double modal group (four from the regionally acceptable group and two from the regionally unacceptable group) and two from the Control group (one each from the regional acceptability groups). The critical regions were identical for the exposure (Phase 1) and test (Phase 2) blocks: the first modal, the critical second modal, and the spillover region, which consisted of the word following the second modal.

Outliers greater than three standard deviations from the mean of the individual regions were again removed. In the exposure block, this resulted in a total data exclusion of 2.4% (1.9% from the first modal region, 2.4% from the second modal region, and 2.8% from the spillover region). In the test block, a total of 2.4% of the data were excluded (2.7% from the first modal region, 2.2% from the second modal region, and 2.2% from the spillover region).

Reading times were analyzed using linear mixed effect models with exposure condition, item order (1–10 in Phase 1, 1–5 in Phase 2), and their interactions as fixed effects and participants as random effects. For each model, the maximal random effect structure that converged was utilized. For the Phase 2 analysis, regional acceptability and its interactions were also included as fixed effects. Exposure condition and regional acceptability were Helmert-coded (double modal group = 1, control group = -1; acceptable = 1, unacceptable = -1). Identical models were used to analyze reading times at each word in the three-word critical region.

### 3.2 Results

#### 3.2.1 Phase 1: Exposure block

**Fig 4:**
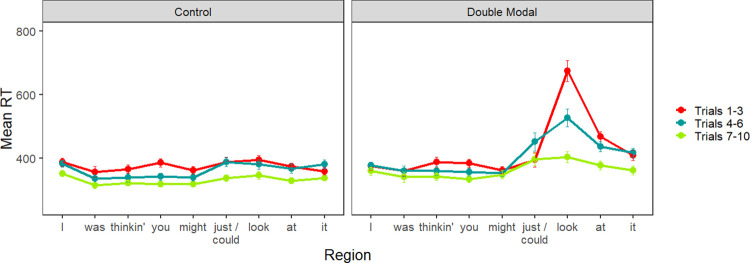
Experiment 2 exposure block. Mean reading times for critical sentence regions by trial number grouped by early (trials 1–3), middle (trials 4–6) and late (trials 7–10). Data from the Control group are on the left, while data from the Double Modal group are on the right.

Word-by-word reading times for the exposure block are summarized in Fig 4. Fig 4 divides the ten critical sentences into "early" (trials 1–3), "middle" (trials 4–7), and "late" (trials 8–10). As expected, reading times increased sharply at the spillover region (*look*), for the first few double modal sentences, then gradually decreased with more exposure.

Table 4 summarizes the statistical analysis for the exposure block; the random effect structure for the two modal regions included only a random intercept, while that of the spillover region also included a random slope for order. In the first and second modal regions (i.e., might and *just/could* in the example sentence), there was an effect of trial order. Participants’ reading times sped up as the experiment progressed, as in Experiment 1. Additionally, there was an interaction between exposure group and order at the second modal, which was caused by the Double Modal group reading this region unexpectedly more slowly in the middle trials compared to the early and late trials. While we did not see a comparable effect in Experiment 1, this may reflect a growing awareness that odd language follows modal verbs. Finally, in the spillover region (look), there was an effect of trial order and an interaction of trial order and exposure group. The interaction in the spillover region indicates that participants in the Double Modal exposure group adapted more strongly than those in the control group, as expected.

**Table 4:** Experiment 2 exposure block fixed effects summary for the three regions of analysis: The first modal region, the critical second modal region, and the spillover region.

	1st Modal Region		2nd Modal Region		Spillover Region	
Predictor	*β*	*SE*	*Β*	*SE*	*Β*	*SE*
Exposure group	2.00	8.23	1.76	13.15	162.94 ***	20.12
Trial order	-5.33 ***	0.78	-4.72 ***	1.33	-24.33 ***	2.14
Exposure x Order	1.28 †	0.78	3.34 *	1.33	-15.92 ***	2.14

Note:

† p < 0.1

* p ≤ 0.05

** p ≤ 0.01

*** p ≤ 0.001

#### 3.2.2 Phase 2: Test block

If the adaptation effects for the Double Modal group during Phase 1 reflect learning of the construction, then the Double Modal group should read acceptable double modals faster than unacceptable double modals, while the Control group should read both acceptable and unacceptable slowly, because this was the Control group’s first exposure to double modals.

**Fig 5:**
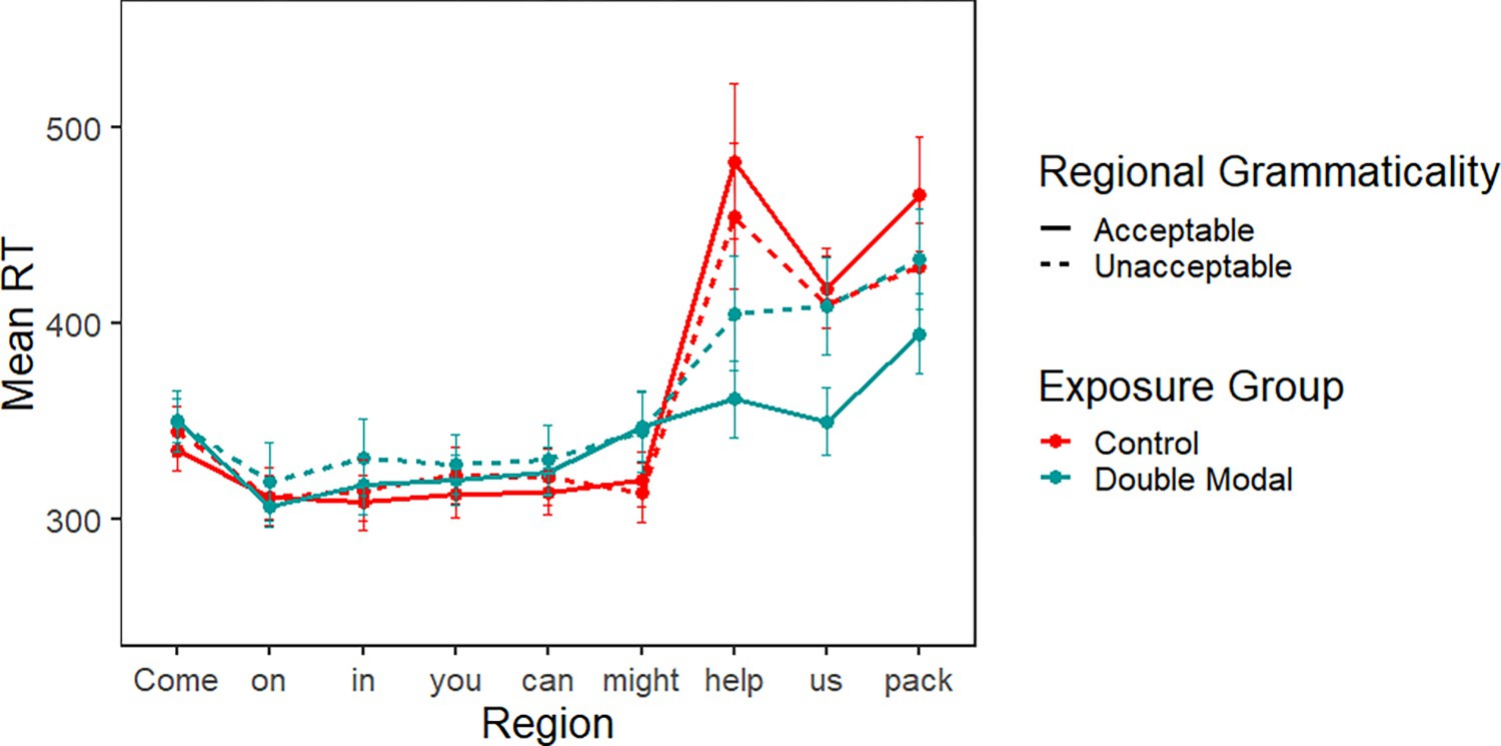
Experiment 2 test block. Mean reading times for critical sentence regions by acceptability of the regional structure (acceptable vs. unacceptable) and exposure group (control vs. double modals). The example sentence is regionally unacceptable; the acceptable version ordered the modals “might can”.

The data pattern is illustrated in Fig 5 and the statistical analysis of the test block is summarized in Table 5; the random effects structure for all three regions was identical and contained only a random intercept. In both the first and second modal regions (i.e., *can* and *might* in the example), there were no significant effects. In the spillover region (i.e., *help* in the example), there was an effect of exposure group; the Double Modal group read this region more quickly regardless of the regional grammaticality of the sentence. As in the Experiment 1, the effect of trial order indicates that participants continued to adapt to the experiment in the test block. Finally, the interaction between exposure group and order reflects the fact that the Control group adapted more strongly during the test phase than the Double Modal group did. Given that this is the Control group’s first exposure to double modal sentences, whether or not they are regionally acceptable, this greater adaptation is not surprising.

**Table 5:** Experiment 2 test block fixed effects summary for the three regions of analysis: The 1st modal region, the critical 2nd modal region, and the spillover region.

	1st Modal Region		2nd Modal Region		Spillover Region	
Predictor	*Β*	*SE*	*Β*	*SE*	*β*	*SE*
Exposure group	7.95	9.69	18.08	12.17	-121.54 ***	22.78
Regional acceptability	-3.47	9.69	-5.12	12.17	-6.65	22.78
Trial order	-0.88	2.30	-1.56	3.15	-27.20 ***	5.37
Exposure x Acceptability	0.48	9.69	6.50	3.15	-4.63	22.78
Exposure x Order	-1.26	2.30	-1.73	3.15	26.21 ***	5.37
Acceptability x Order	0.10	2.30	3.39	3.15	0.58	5.37
Exposure x Acceptability x Order	0.22	2.30	-1.82	3.15	-3.93	5.37

Note:

† p < 0.1

* p ≤ 0.05

** p ≤ 0.01

*** p ≤ 0.001

An interaction between exposure and acceptability would have indicated that the Double Modal group had learned about the lexical patterning of double modals from concentrated exposure to regionally acceptable usages of the construction during Phase 1. The absence of the predicted interaction suggests that participants did not learn the pattern. As in Experiment 1, we calculated Bayes factors for the spillover region to evaluate evidence for the null.

#### 3.2.3 Bayes analysis

First, we calculated Bayes factors examining whether exposure group had any effect at all on behavior in the test phase by comparing the full model to a model without the exposure group factor and its interactions. The BF of 13660.94 ±0.16% indicates extreme evidence for the full model, reflecting that participants who had been in the Control group read both conditions slower than the Double Modal group in the spillover region. Next, we compared the full model to a model missing the exposure by acceptability interaction, to test whether these results indicate a lack of learning the lexical patterning of the double modal construction. We found moderate evidence for the null hypothesis, BF of 0.14 ±0.26%, i.e., the construction was not learned. Finally, we conducted a direct within-group comparison of the full model to a model without the regional grammaticality factor and its interactions. For the Control group, the BF of 0.06 ±0.82% indicates strong evidence for the null hypothesis. Similarly, the BF of 0.20 ±0.5% indicates moderate evidence for the null for the Needs group. In other words, the regional grammaticality factor was not needed to describe the data for either group.

### 3.3 Discussion

There were three important findings. First, we found robust adaptation effects (decreasing reading times) across the first ten exposures to the double modal construction. This replicates similar effects found for the *needs* construction, both in Experiment 1, and in prior research using unrelated *needs* sentences as stimuli [1, 2]. This finding extends the adaptation paradigm to a second regional construction.

Second, readers who adapted to acceptable double modal constructions during Phase 1 did not reliably distinguish between double modals that occurred in the order that they were exposed to (*must/may* in the first position) and double modals that followed a new pattern (*must/may* in the second position).

Third, in contrast to Experiment 1, participants who had been exposed to the regional construction during Phase 1, had faster reading times in the spillover region of Phase 2 compared with the Control group, without regards to the regional grammaticality of the construction. When comparing the reading time pattern in the spillover regions of Figs 2 and 5, we see that although the patterns are very similar, the magnitude of the difference between the Control group and the Double Modal group (Fig 5) is considerably larger than the comparable contrast in Fig 2. The greater magnitude of difference may reflect that the Control group in Experiment 2 found the double modal stimuli to be more unusual than the Experiment 1 Control group found the *needs* sentences.

We also noted that the overall morphology of the adaptation patterns appeared to differ in Experiments 1 and 2, based on a comparison of Figs 1 and 4. The most interesting difference is that reading times at the critical region appeared to flatten out more quickly in Experiment 1, compared with Experiment 2. The figures seem to suggest that adaptation to the regional construction occurred more gradually in Experiment 2 compared with Experiment 1. It is unclear whether this is due to differences between the regional constructions, the stories, or the participants.

Experiment 2 demonstrated that adaptation to regional syntactic constructions during reading is robust in some respects and not so robust in others. This type of implicit learning is robust in the sense that the effects on reading time can be rapid and dramatic, and occur not only for the *needs* construction, but for another regional construction as well. However, the representations that are learned from brief exposure through reading—even when the construction is learned via felicitous dialogue—appear to be more general than the representations learned natively in real-life interactions.

Previous work on the *needs* construction found that adaptation effects generalized to other felicitous forms of the construction, suggesting that learning was not superficial [1, 2]. However, our results indicate that generalization of adaptation effects do not themselves demonstrate acquisition of the dialect-internal grammaticality conditions for a novel construction. Neither Experiment 1 nor Experiment 2 found evidence that adaptation led to learning basic grammatical information about the construction, such as verb tense (*needs* construction) or the ordering of modal verbs (double modal construction). While the results of Experiment 1 were somewhat equivocal, Experiment 2 provided strong evidence for the null hypothesis.

## 4. Experiment 3

The goals of Experiment 3 were threefold. First, we expected to replicate the adaptation pattern found for the *needs* construction in Experiment 1, using both stories from Experiment 1 together for the exposure phase. Using both stories increases the amount of exposure from nine tokens to 15 tokens, potentially allowing for better learning of the construction’s grammar. Second, we tested construction learning in a new way; we measured construction knowledge more directly by collecting explicit acceptability judgments during the test phase.

### 4.1 Method

#### 4.1.1 Participants

Forty-eight participants from the University of Michigan Psychology Subject Pool completed the experiment for partial course credit. One additional participant was run but was excluded for poor attention and excessive speed in the self-paced reading portion of the experiment. Fewer participants were recruited for this study compared with Experiments 1 and 2, because there were only two participant groups, not four.

#### 4.1.2 Materials

Participants read two narratives (35 sentences) one word at a time. Interspersed were 15 target sentences. These narratives were taken from Experiment 1, except that the second story followed the same pattern of *needs* exposure as the first. Participants assigned to the Needs group read *needs* constructions in all 15 target sentences. Participants assigned to the Control Group read versions of these sentences that are acceptable in SAE.

The critical stimuli for the acceptability judgment portion of this experiment consisted of 18 sets of sentences with different forms of *needs*: a version that is acceptable in SAE (5a), one that is acceptable in the Regional Dialect (RD, 5b), and one that is ungrammatical in both (5c). The ungrammatical sentences always consisted of *need(s)* followed by the bare form of the verb rather than the past participle, which is not an acceptable form of the *needs* construction. These 18 items were counterbalanced across three lists, so that each participant saw only one version of each target item. Eighteen filler items were constructed from other regional phenomena found through the Yale Grammatical Diversity Project [27]. The critical items from the double modal version of this experiment (see Experiment 4) served as an additional 18 fillers for a total of 36 fillers. These fillers were randomly interspersed with the target items for a total of 54 items. Stimuli from the acceptability judgment task, including the fillers, are given in S2 Appendix.

(5) a. Acceptable in SAE: The term paper **needs to be revised** before tomorrow morning.
b. Acceptable in RD: The term paper **needs revised** before tomorrow morning.
c. Ungrammatical: The term paper needs revise before tomorrow morning.

#### 4.1.3 Procedure

The experiment was built using IbexFarm [28], and participants were tested remotely while supervised by an experimenter via a Zoom window. After reading and signing an informed consent form, participants were assigned to one of two exposure groups; the Needs group was exposed to the *needs* construction during the self-paced reading portion of the study, while the control group read the SAE counterparts of these constructions. In the exposure phase, participants completed a self-paced reading task, similar to that from Experiment 1. The procedure was nearly identical except that the second story was included in the exposure block and, therefore, did not test participants’ knowledge of the *needs* construction.

In the test phase of the experiment, participants completed an acceptability judgment task. They were instructed to rate sentences on a Likert scale from 1 (bad) to 7 (good). As part of the instructions, participants were provided with detailed directions and examples to illustrate that the task is not about stylistic considerations, prescriptive norms, or the plausibility of the described event, but rather about what they could imagine someone saying in conversation. This included examples with varying degrees of acceptability to illustrate sentences that correspond to different values on the scale. None of the example sentences included regional constructions.

Additionally, the first six experimental trials were identical for all participants; these served as a pre-test, calibration phase. These trials consisted of two highly acceptable sentences, two highly unacceptable sentences, and two marginal sentences. This calibration portion was designed to encourage participants to use the entire scale. The use of the entire scale is particularly important for this experiment because the items are all taken from regional dialects. For example, if participants limited their judgments to the extreme ends of the scale, they may rate all the sentences as unacceptable if they are not speakers of any of the dialectal features that are included in the items. As we are interested in more subtle differences between acceptable regional constructions and unacceptable versions of those constructions, a non-binary judgment was required.

#### 4.1.4 Analysis

The analysis of the self-paced reading data was very similar to that from the exposure phase (Phase 1) of Experiment 1. Errors in item ten were corrected for this experiment, and it is therefore included in this analysis. As in prior experiments, participants whose accuracy on the comprehension questions was less than two standard deviations below the mean were dropped from the analysis (i.e., accuracy less than 75%), which excluded two participants from the Needs group and two participants from the Control group (four total). The critical regions were the same: *need(s)*, the critical verb, and the spillover region, consisting of the word following the critical verb. The outlier removal procedure was identical–reading times greater than three standard deviations from the mean of the individual regions were removed–which led to a total data exclusion of 1.3% (1.2% from the *need(s)* region, 2.0% from the critical verb region, and 0.8% from the spillover region).

As in prior experiments, reading times were analyzed using linear mixed effect models. Exposure group, item order, and their interaction were the fixed effects in Experiments 3 and 4. To analyze the acceptability judgements, the raw ratings were first converted to *z*-scores within participants including both targets and fillers, following Schütze and Sprouse [29]. This transformation converts a participant’s scores to units representing the number of standard deviations difference an individual rating was from that participant’s mean rating. *Z*-scoring standardizes the results to the same scale, thus correcting for any differences between participants’ use of the scale (e.g., not all participants may have used all seven available points on the Likert scale).

The data were analyzed using linear mixed-effect models. Two fixed effect factors were used to compare the three levels of acceptability. The first compared the grammatical SAE sentences to those that would be labeled as ungrammatical in SAE, which included both the regionally acceptable sentences and the ungrammatical sentences; this factor was Helmert coded (grammatical SAE = 2/3, acceptable in RD and ungrammatical = -1/3). The second directly compared the regionally acceptable sentences to their ungrammatical counterparts (grammatical RD = 1, ungrammatical = -1).

### 4.2 Results

#### 4.2.1 Self-paced reading

**Fig 6:**
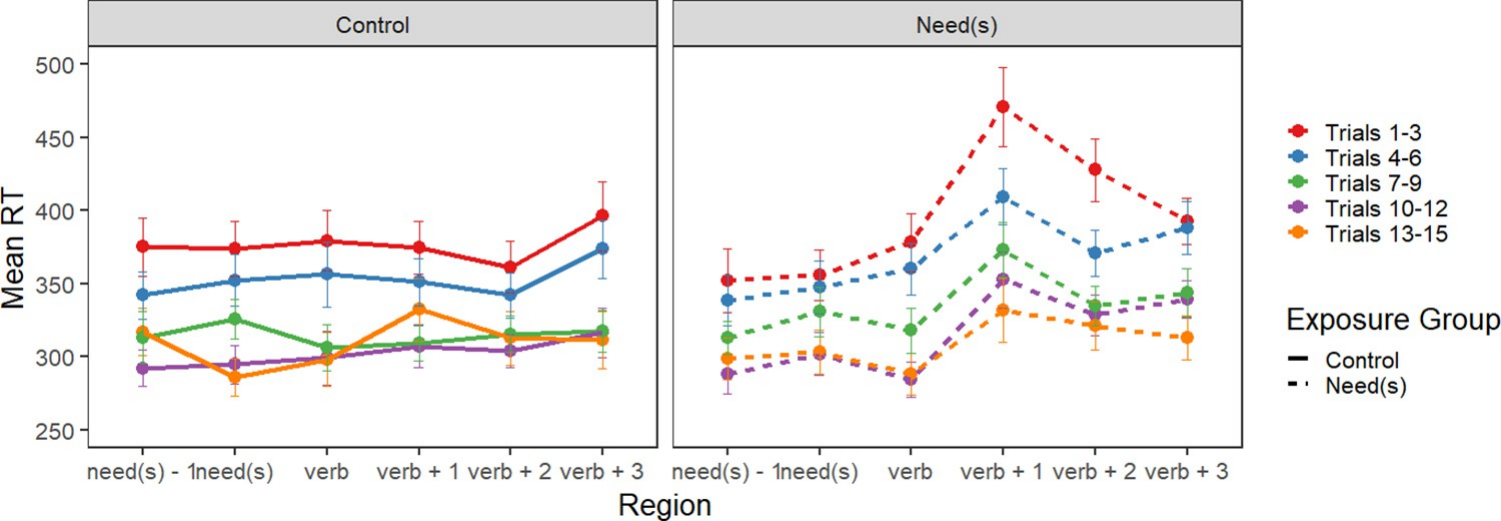
Experiment 3 exposure block. Mean reading times for critical sentence regions by trial number grouped in bins of 3 trials. Data from the control group are plotted on the left, while that from the *needs* exposure group are plotted on the right.

Word-by-word reading times for the exposure phase of the experiment are plotted in Fig 6. Darker colors indicate earlier trials, while lighter colors indicate later trials.

The statistical analysis of the self-paced reading data is summarized in Table 6. In all three regions, there was an effect of trial order; participants’ reading times decreased across the experiment in both conditions, as in prior experiments.

**Table 6:** Experiment 3 self-paced reading fixed effects summary for the three regions of analysis: The *need*(s) region, the critical verb region, and the spillover region.

	*need(s)* Region		Verb Region		Spillover Region	
Predictor	*β*	*SE*	*β*	*SE*	*β*	*SE*
Exposure group	-9.76	15.00	4.89	16.83	53.95 **	18.52
Trial order	-5.86 ***	0.81	-7.56 ***	0.79	-7.74 ***	1.25
Exposure x Order	1.29	0.81	-0.59	0.79	-3.34 **	1.25

Note:

† p < 0.1

* p ≤ 0.05

** p ≤ 0.01

*** p ≤ 0.001

In the spillover region (i.e., verb + 1), the Needs group had longer reading times overall and there was the expected interaction between exposure group and trial order, with the Needs group decreasing their reading times more than the Control group did.

#### 4.2.2 Acceptability judgments

**Fig 7:**
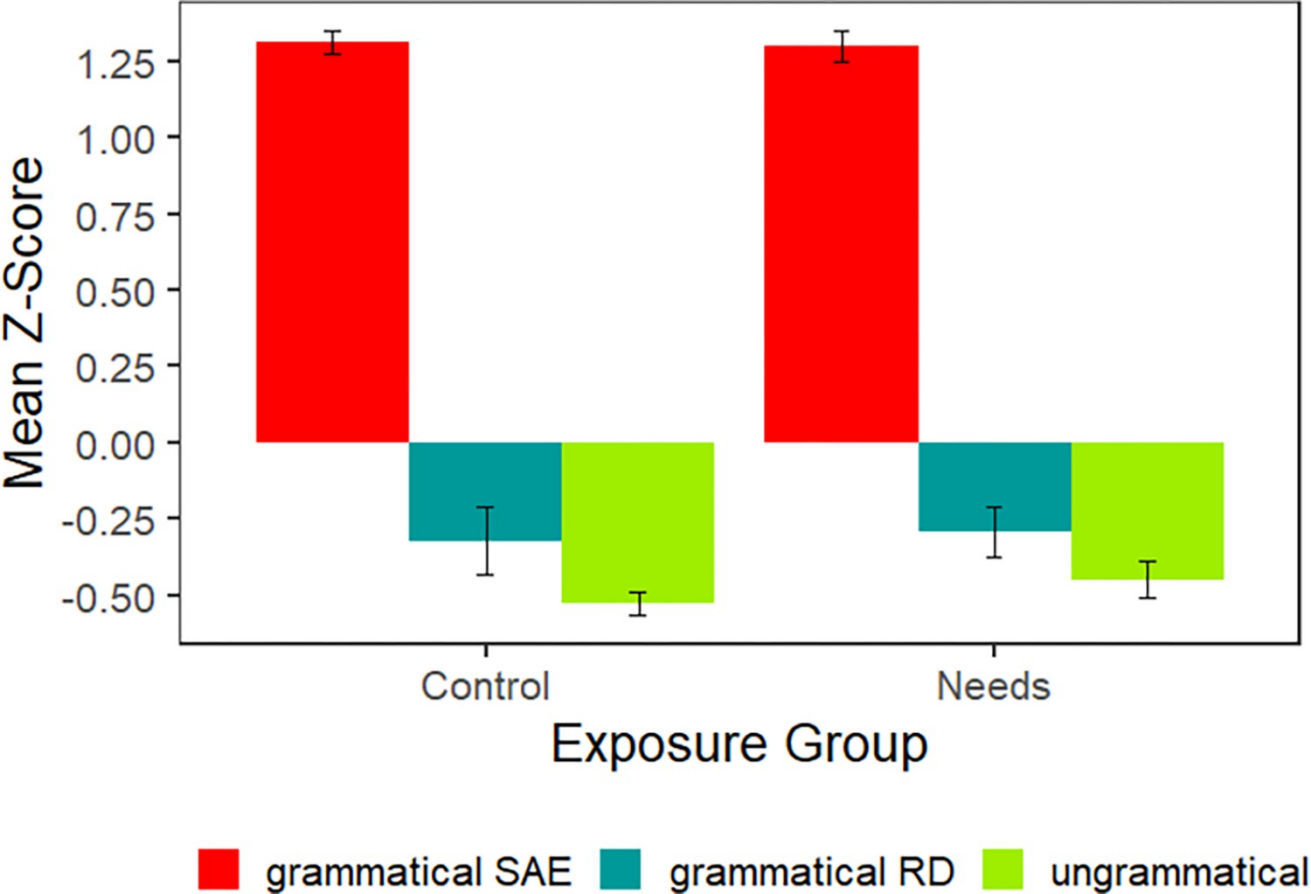
Experiment 3 mean *z*-scores. Plotted by exposure group for acceptable sentences in Standard American English (*The lawn needs to be mowed*.), acceptable sentences in the regional dialect (*The lawn needs mowed*.), and unacceptable sentences in either (*The lawn needs mow*).

The acceptability judgments are plotted in Fig 7. As expected, both exposure groups rated the sentences that are grammatical in SAE highly. The mean ratings for both of the regional sentences were below zero, on the ungrammatical side of the scale.

Table 7 summarizes the analysis of participants’ *z*-scores. Sentences that were grammatical in SAE were rated significantly higher than the other two conditions. In addition, regionally grammatical sentences were rated higher than sentences that were ungrammatical in both SAE and regional dialects. Crucially, there was no interaction between exposure group and judgments for grammatical sentences in the regional dialect and ungrammatical sentences. In other words, participants exposed to the *needs* construction did not rate regionally acceptable sentences more highly than unacceptable ones.

**Table 7:** Experiment 3 acceptability fixed effects summary.

Predictor	Β	SE
Exposure group	0.02	0.04
Grammatical SAE vs. Rest	1.70 ***	0.06
Grammatical RD vs. Ungrammatical	0.09 *	0.04
Exposure x Gram SAE vs. Rest	-0.03	0.06
Exposure x Gram RD vs Ungram	-0.01	0.04

Note:

† p < 0.1

* p ≤ 0.05

** p ≤ 0.01

*** p ≤ 0.001.

Grammatical SAE vs. Rest represents the factor comparing the grammatical SAE sentences to the other two conditions (grammatical RD and ungrammatical). Grammatical RD vs. Ungrammatical represents the factor directly comparing the regionally acceptable sentences (grammatical RD) to their ungrammatical counterparts.

#### 4.2.3 Bayes analysis

As in the previous experiments, we want to attribute the fact that the exposure groups respond in similar ways to grammatical RD and ungrammatical sentences to a lack of learning (although in this instance, both groups demonstrate an understanding that the *needs* construction is more acceptable than the ungrammatical sentences). As in the first two experiments, we calculated a Bayes factor comparing the full model to a model without the exposure group factor or its interactions; the BF of less than 0.001 ±21.39% indicates that there is extreme evidence for the null, i.e. exposure group did not impact acceptability judgments. Nonetheless, we again calculated a Bayes factor for our more specific hypothesis: if the Needs group had truly learned the *needs* construction during the exposure phase, the Needs group, and only this group, should have reliably rated the grammatical RD sentences more highly than the ungrammatical sentences. Thus, we calculated a Bayes factor comparing the full model to a model lacking the critical interactions between exposure group and both grammaticality comparisons; the BF of 0.02 ±33.35% indicates very strong evidence for the null hypothesis. The Needs group responded to the regionally grammatical sentences in the same way that the Control group did, which suggests that the difference in these conditions was due to factors other than learning (e.g., familiarity with the structure in popular culture).

To further support this claim, we ran Bayes analyses for each individual exposure group comparing the full model with a null model that does not include the factor comparing regionally grammatical constructions to ungrammatical ones (Grammatical RD vs. Ungrammatical). For both groups, the BFs indicate moderate evidence for the null model (Needs: 0.28 ±0.95%, Control: 0.33 ±0.89%). This, again, suggests that neither group requires the grammatical RD versus ungrammatical factor to explain the data.

### 4.3 Discussion

The reading time data replicated and extended the results of Experiment 1, which also investigated adaptation to the *needs* construction. In Experiment 1, we observed adaptation across nine tokens of the *needs* construction. In this experiment, the adaptation phase included 15 occurrences (see Fig 7, Needs panel). In contrast to Experiment 1, in which very long reading times at the verb+1 condition were limited to the first three trials for the Needs group, relatively slow reading times at verb+1 persisted across the exposure phase in Experiment 3, more like the pattern observed in Experiment 2 for the DM group. Given that the stimuli were the same in Experiments 1 and 3, this apparent difference in the rate of adaptation is likely due to either the remote data collection or some difference in the participants. Based on the familiarity data in S4 Appendix (Figs D1 vs. D3), this participant sample was somewhat more familiar with the construction than the sample from Experiment 1. Despite these differences, the predicted adaptation effects were replicated.

The crucial question is whether exposure to the 15 examples of the *needs* construction in a realistic context leads to sufficient grammatical knowledge of the construction’s verb tense constraints to distinguish between regionally acceptable forms of the *needs* construction and ungrammatical variants. The answer was “no.” Participants in both exposure groups rated the *needs* construction as ungrammatical, as measured by our primary analysis of the judgment data and by the targeted Bayes analysis. In both Bayes analyses, there was extreme evidence for the null (no learning) hypothesis. These findings add to the evidence from Experiments 1 and 2 that adaptation effects do not entail, and may not be characterized by, learning the grammar of a regional construction.

Interestingly, both exposure groups rated the regionally acceptable *needs* construction (*needs revised*) as less unacceptable than the ungrammatical condition (*needs revise*). This mirrors a statistical pattern in English. The regional construction is a present tense verb followed by a past participle verb and this pattern is relatively common (e.g., *get started*, *feels obligated*, *become enamored*, *is expected*). The Corpus of Contemporary English [30] contained 2,647,118 tokens of this type, with 8,767 unique combinations in December, 2022. There are also examples that are similar to the ungrammatical form, a present tense verb followed by the unaffixed infinitive form (e.g., *helps prevent*, *does try*). However, this pattern is much less common, with only 79,487 tokens and 1,129 unique combinations.

A potential limitation of the acceptability judgments is that the *needs* exemplars during the exposure phase occurred in quoted speech within a story, whereas the items in the acceptability judgment task appeared as isolated sentences without attribution to a particular speaker, or any other type of story context. We anticipated this issue, and formulated our instructions to mitigate it. Participants were instructed to ignore stylistic considerations and prescriptive norms. Instead participants were asked to imagine someone saying each sentence in conversation, and evaluate it in that context. Despite these instructions, we acknowledge that participants bring their own language attitudes to the study and that their evaluations of stimuli are influenced to some degree by prescriptive feedback that they have received in the past.

## 5. Experiment 4

This experiment is parallel to Experiment 3, except that it uses the double modal stimuli from Experiment 2. The goals of this study mirror those of Experiment 3.

### 5.1 Method

#### 5.1.1 Participants

Forty-eight participants from the University of Michigan Psychology Subject Pool completed the experiment for partial course credit.

#### 5.1.2 Materials

As in Experiment 3, participants read two narratives (35 sentences) one word at a time. Interspersed were 15 target sentences. These narratives were taken from Experiment 2, except that the second story followed the same pattern of double modal exposure as the first. Participants assigned to the Double Modal Group read sentences with double modals in all 15 target sentences, while those assigned to the Control Group read versions of these sentences that are acceptable in SAE.

As in Experiment 3, the stimuli for the acceptability judgment portion of this experiment consisted of 18 sets of sentences with different forms of the double modal: a version that is acceptable in SAE (6a), one that is acceptable in the Regional Dialect (6b), and one that is ungrammatical in both (6c). The ungrammatical sentences always consisted of the two modals in the regionally acceptable sentence in the reverse order (e.g., *should might* instead of *might should*), which is unacceptable to speakers of this dialect. These 18 items were counterbalanced across three lists, so that each participant saw only one version of each target item. The same 18 filler items from Experiment 3 that were constructed from other regional phenomena were also used in this experiment. The critical items from Experiment 3 (i.e., the *needs* construction version of this experiment) served as an additional 18 fillers for a total of 36 fillers. These fillers were randomly interspersed with the target items for a total of 54 items. Stimuli from the acceptability judgment task, including the fillers, are given in S2 Appendix.

(6) a. Acceptable in SAE: You **should** eat before you go to work.
b. Acceptable in RD: You **might should** eat before you go to work.
c. Ungrammatical: You **should might** eat before you go to work.

#### 5.1.3 Procedure

The procedure was identical to that of Experiment 3, except that participants were randomly assigned to either the Double Modal or Control exposure group. The Double Modal group was exposed to the double modal construction during the self-paced reading portion of the study, while the Control group read their SAE counterparts. The second portion of the study–the acceptability judgement task–was identical to that in Experiment 3.

#### 5.1.4 Analysis

The analyses of the self-paced reading and acceptability judgment data in this experiment were identical to that of Experiment 3. As in prior experiments, participants whose accuracy on the comprehension questions was less than two standard deviations below the mean were not included in the analysis (i.e., their accuracy was less than 80%); two participants were dropped, both from the Double Modal Group. The critical regions were the same as those in Experiment 2: the first modal, the critical second modal, and the verb. The outlier removal procedure was identical as well–reading times greater than 3 standard deviations from the mean of the individual regions were removed–which led to a total data exclusion of 1.4% (1.8% from the first modal region, 1.6% from the critical second modal region, and 2.1% from the verb region).

### 5.2 Results

#### 5.2.1 Self-paced reading

**Fig 8:**
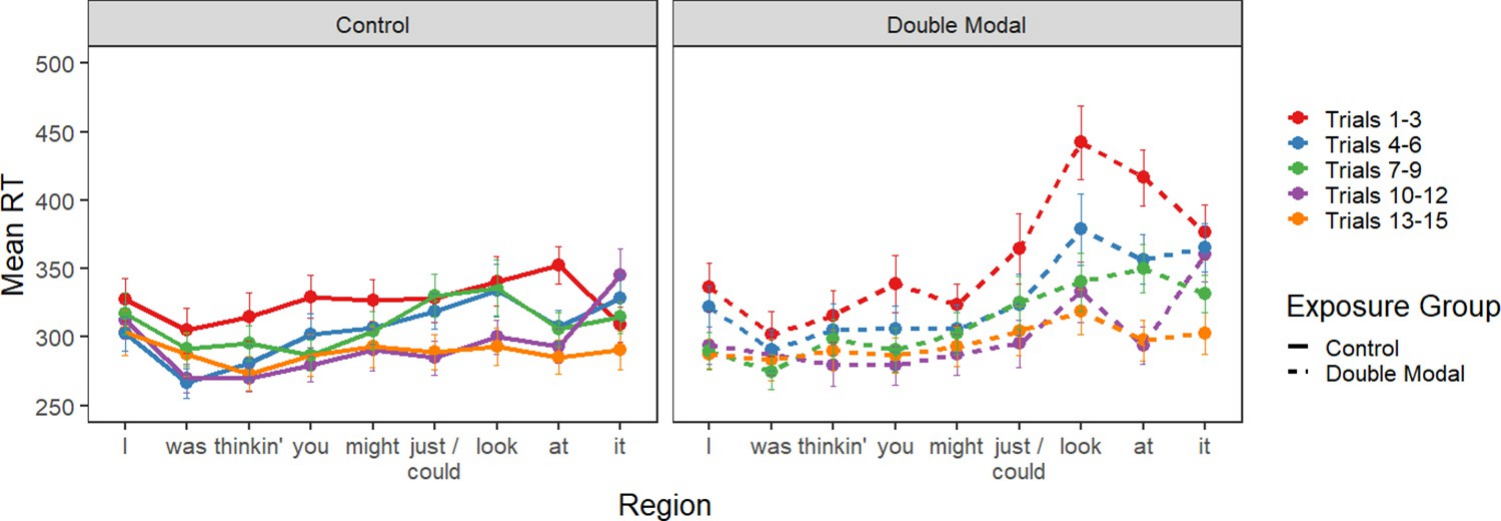
Experiment 4 exposure block. Mean reading times for critical sentence regions by trial number grouped in bins of 3 trials. Data from the control group are plotted on the left, while that from the double modal exposure group are plotted on the right.

Word-by-word reading times for the exposure phase of the experiment are plotted in Fig 8. Darker colors indicate earlier trials, while lighter colors indicate later trials. In general, participants’ reading times decreased as the number of trials increased, particularly for those in the Double Modal group in the region just after the critical second modal (i.e., the spillover region, *look* in the example sentence).

Table 8 summarizes the linear mixed effect models for the exposure phase. In all three regions, there was an effect of the order of trials; later trials were read more quickly than earlier ones, as in prior experiments. In the spillover verb region, there was an effect of exposure group, with longer reading times for participants in the Double Modal group. Crucially, we found the expected interaction of exposure group and trial order, which indicated that the Double Modal group demonstrated greater decreases in reading time in this region than the Control group.

**Table 8:** Experiment 4 self-paced reading fixed effects summary for the three regions of analysis: The 1st modal region, the critical 2nd modal region, and the spillover region.

	1st Modal Region		2nd Modal Region		Verb Region	
Predictor	*Β*	*SE*	*Β*	*SE*	*β*	*SE*
Exposure group	-0.39	14.72	12.17	17.63	41.36 *	19.32
Trial order	-3.08 ***	0.70	-4.50 ***	0.81	-7.31 ***	0.94
Exposure x Order	0.07	0.70	-0.54	0.81	-2.50 **	0.94

Note:

† p < 0.1

* p ≤ 0.05

** p ≤ 0.01

*** p ≤ 0.001

#### 5.2.2 Acceptability judgments

**Fig 9:**
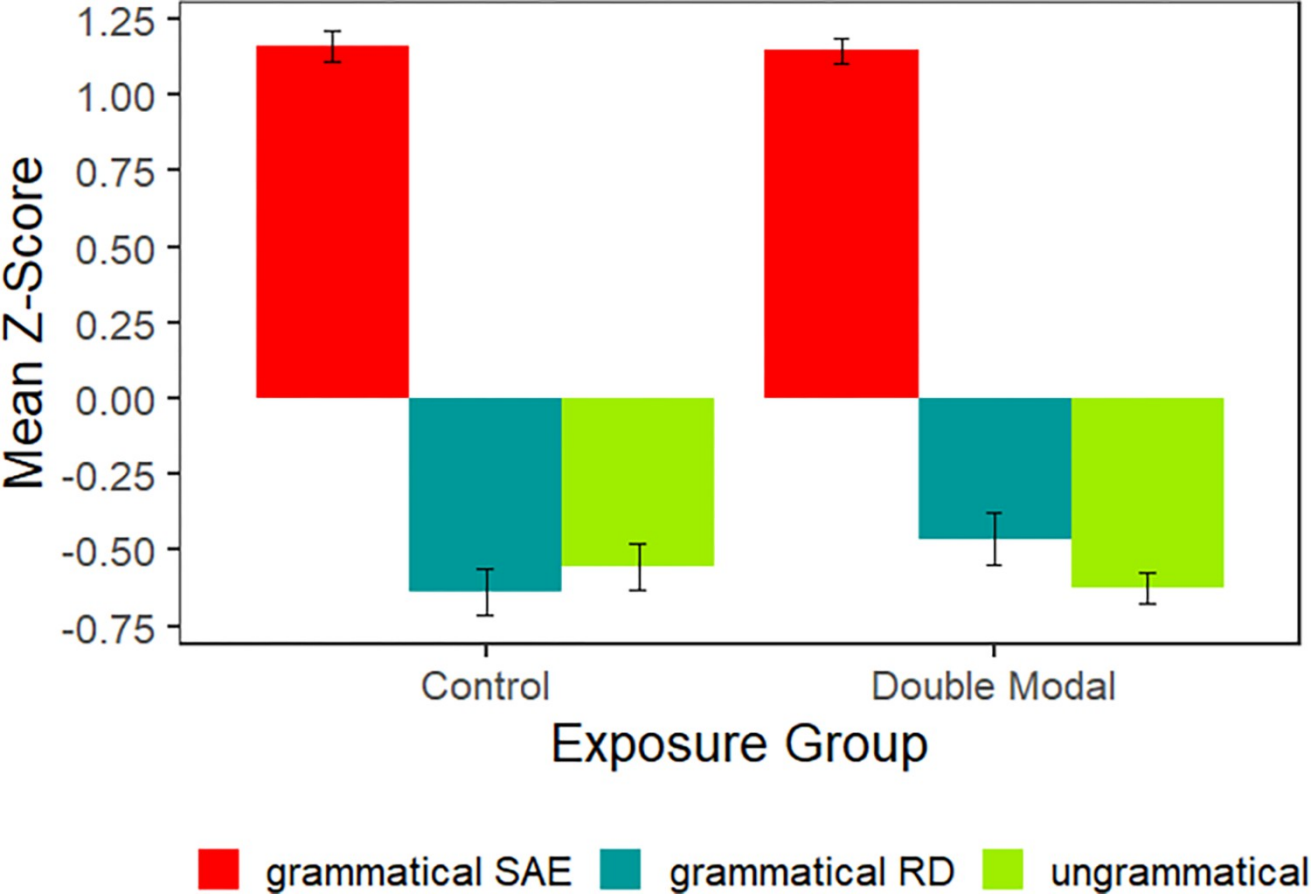
Experiment 4 mean *z*-scores. Plotted by exposure group for acceptable sentences in Standard American English (*She should go to work*), acceptable sentences in the regional dialect (*She might should go to work*), and unacceptable sentences in either (*She should might go to work*).

The results of the acceptability portion of the experiment are plotted in Fig 9. As in Experiment 3, participants assigned high ratings to sentences that are grammatical in SAE and low ratings to those that are ungrammatical in SAE (i.e., those that are grammatical in the regional dialect and ungrammatical in both SAE and the regional dialect).

The statistical analysis of the acceptability judgment portion of the study is summarized in Table 9. Unsurprisingly, sentences that were grammatical in SAE were rated higher than the two conditions that are ungrammatical in SAE. The learning hypothesis predicted an interaction between Exposure and the factor, “Gram RD vs Ungram”, but no such interaction was observed.

**Table 9:** Experiment 4 acceptability fixed effects.

Predictor	*β*	*SE*
Exposure group	0.002	0.05
Grammatical SAE vs. Rest	1.72 ***	0.07
Grammatical RD vs. Ungrammatical	0.02	0.04
Exposure x Gram SAE vs. Rest	-0.03	0.07
Exposure x Gram RD vs. Ungram	0.06	0.04

Note:

† p < 0.1

* p ≤ 0.05

** p ≤ 0.01

*** p ≤ 0.001

“Grammatical SAE vs. Rest” is the factor comparing the grammatical SAE sentences to the other two conditions (grammatical RD and ungrammatical). “Grammatical RD vs. Ungrammatical” is the factor comparing the regionally acceptable sentences (grammatical RD) to their ungrammatical counterparts

#### 5.2.3 Bayes analysis

As with the previous experiments, we calculated a Bayes factor comparing the full model to a model lacking the exposure group factor and its interactions; the BF of 0.001 ±2.77% indicates extreme evidence for the null hypothesis. That is, exposure to 15 double modal sentences did not impact the participants’ acceptability judgments. To be comprehensive, we again calculated a Bayes factor for our more specific hypothesis that if the Double Modal group had truly learned the double modal construction during the exposure phase, they (and not the Control group) should have reliably rated the grammatical RD sentences more highly than the ungrammatical sentences. Thus, we calculated a Bayes factor comparing the full model to a model lacking the critical interactions between exposure group and both grammaticality comparisons; the BF of 0.02 ±2.89% indicates very strong evidence for the null hypothesis. Finally, pairwise by exposure group comparisons of the full model to null model without the grammatical RD versus ungrammatical factor also indicate evidence for the null. For the Double Modal group, the BF of 0.19 ±0.9% is moderate evidence for the null, while the BF of 0.10 ±0.85% for the Control group is strong evidence for the null. Again, the regionally grammatical versus ungrammatical comparison is not needed to explain these data.

### 5.3 Discussion

Experiment 4 replicated and extended upon the adaptation effects observed in Experiment 2, with reading exposure to double modals. The additional exposures (15 vs 10 in Experiment 2) provided additional opportunity for learning the construction, and in particular, the consistent ordering of the modal verbs. The 15 exposures included twelve examples of *might* as the first modal, and two examples of *may* as the first modal (See S2 Appendix). Neither *might* nor *may* ever occurred as the second modal. Even so, participants judged the acceptability of sentences containing *could might* as equivalent to *might could*, regardless of whether they had been in the Double Modal group or the Control group. Exposure during the experiment did not impact acceptability in either the primary analysis or the Bayes factor analyses. In fact, the Bayes analyses yielded consistent evidence for the null hypothesis.

## 6. General discussion

In all four experiments, participants exposed to a regional construction exhibited robust adaptation effects, but they did not demonstrate knowledge of that construction compared to the control groups, either via explicit acceptability judgments (Experiments 3 and 4) or via distinctive reading time patterns for regionally acceptable vs unacceptable sentences (Experiments 1 and 2). A similar disjunction between reading time data and acceptability judgments has recently been reported [31]. Understanding what exactly is learned in adaptation experiments is crucial, both for knowing how to interpret prior results, and for effectively leveraging experimental paradigms in future research.

Prior reports of adaptation effects presented novel or unusual constructions in lists of isolated sentences [1–6]. This could have hampered grammatical learning because this context is very unlike the contexts in which regional constructions are encountered naturally. In the four experiments presented here, we tried to optimize the learning context, while sticking with the self-paced reading paradigm, by embedding the regional constructions in stories containing casual dialogue and set in environments where the constructions might naturally occur. Even so, no evidence for grammatical learning could be found. Rather than acquiring structural knowledge beyond the surface form, our data indicate that readers failed to learn the surface form.

The conclusion cannot be that adults do not acquire new grammatical constructions when exposed to regional variation; clearly this does happen in real life experience. Rather, the current data indicate that the reading time adaptation paradigm is not well suited to investigate the acquisition of grammatical constructions.

There are at least two possible accounts of the difference between mature users of regional constructions and the nascent users trained via text exposure in the current experiments and similar prior studies. Each account must explain both the adaptation effects and the finding that readers trained on a particular construction also generalize to other variants of the construction [1, 2]. It is these generalization effects that have supported claims that adaptation effects reflect real grammatical learning.

On the first account, exposure to a novel grammatical construction opens the door to a new form-to-meaning mapping, which is not yet solidified. During this early stage of adult syntactic learning, it might be advantageous to adopt a relatively unconstrained representation of the form, which then becomes more constrained with experience. This account maintains that the adaptation effects reflect true grammatical learning, in its early stages. What isn’t clear is whether continued input of the same sort would mature into grammatical knowledge that includes specifications for verb tense and word order, and if so, how much input would be required. In the current study, fifteen tokens of a regional construction were insufficient to learn relatively simple grammatical rules about verb tense (*needs* construction) and the ordering pattern of modal verbs (double modal construction). A slightly different version of this account is that in order to progress to more detailed grammatical knowledge, the adult would need to be exposed to the construction in a wider variety of contexts. This family of accounts is plausible, but feels incomplete without greater specification of a learning model.

On the second account, repeated exposure to an unfamiliar construction leads participants to expect all varieties of ungrammatical sentences in the experimental context, essentially dismissing the linguistic input as untrustworthy, with no changes to the participants’ mental grammar. On this account, the adaptation paradigm is of no use for investigating acquisition of a new grammatical construction.

A third account is consistent with the research reported here, but not the generalization effects reported elsewhere. Perhaps exposure to regional variants in this reading paradigm has no effect on readers’ syntactic knowledge because they process the regional variants as errors, which the readers understand by mentally correcting the regional forms to a more familiar form. On this account, adaptation effects reflect increasing efficiency in processing the errors, as they encounter similar errors repeatedly. Under one version of this account, the reader learns to expect that a particular speaker will repeatedly make these errors, but that expectation should not impact acceptability judgments [32]. Either way, this account is unable to explain the generalization effects observed in prior studies [1, 2].

Both of the methods used here to test for grammatical learning have some limitations. In Experiments 1 and 2, we looked for a difference in reading times between acceptable and unacceptable variants of the regional construction after nine or ten exposures. This required us to use a between groups design that lowered our statistical power. We compensated for this by recruiting large numbers of participants, but it is possible that we could have detected a learning effect in the test block of Experiments 1 and 2 with a larger sample, given that there were non-significant numerical differences in the direction predicted by the learning hypothesis, and high variability in reading time during the critical region. This possibility motivated both the Bayes Factor analyses of the test block data in Experiments 1 and 2, and the use of acceptability judgments in Experiments 3 and 4.

Although there are many examples of using acceptability judgments to measure the acquisition of artificial grammars [33, 34], acceptability judgments can be contaminated by prescriptivist grammar attitudes, making it difficult to measure acceptability of constructions that are not part of SAE. To guard against this problem we used instructions that asked participants to imagine someone producing the sentences during the acceptability task. These instructions were successful, in that participants rated the regionally grammatical *needs* sentences higher than fully ungrammatical variants in Experiment 3. However, these findings were not replicated with the double modal acceptability judgments in Experiment 4.

In sum, despite the reported evidence for syntactic generalization following adaptation to a novel construction via self-paced reading [1, 2], it is unlikely that reading-based adaptation paradigms result in knowledge of the construction’s surface form. Such construction learning may be possible through reading examples alone, given enough input, but thus far there has been no evidence of it.

## Supporting information

S1 Appendix Experiments 1 & 3 reading time stimuli. (PDF)

S2 Appendix Experiments 2 & 4 reading time stimuli. (PDF)

S3 Appendix Acceptability judgment stimuli from Experiments 3 & 4. (PDF)

S4 Appendix Construction familiarity responses. (PDF)
