# Supplementary material for: What do we learn when we adapt to reading regional constructions?
Source: PLoS One. 2023 Apr 7;18(4):e0282850. doi: 10.1371/journal.pone.0282850 (PMC10081778; doi:10.1371/journal.pone.0282850)
Supplement: S1 Appendix — (PDF) [file pone.0282850.s001.pdf]

Appendix A. Experiments 1 & 3 Reading Time Stimuli

Experiments 1 and 3 utilized the same two stories. The stories are separated below by a horizontal line. Comprehension questions appear at positions in the story where they appeared during the experiment. In the version below, both stories contain tokens of the *needs* construction (underlined here, but not in the experiment) that are regionally acceptable. Participants in the Control group read a version of the first story in which *to be* was inserted between *need(s)* and the past participle verb. In the ungrammatical version of the second story, the participle verb was replaced by a bare verb, unmarked for tense or number.

Susan finished packing her suitcase and waited nervously with a cup of coffee for Bob to arrive.
He was a college student who she'd hired to housesit while she was gone for a few days.
He needs to get here soon, she thought, I gotta get gas on the way to the airport.
  Did Susan drink tea? (no)
  Is Bob a high school student? (no)
When Bob finally showed up, Susan went over the list she had made.
"I've already fed the dog today, but he will <u>need fed</u> every morning around 8:00.
Then stand back—he really wolfs it down," she warned.
He will also <u>need walked</u> in the mornings and the evenings."
Bob smiled, "Dogs are so awesome! What's his name?"
Susan returned the smile. "Max. Just don't teach him any bad habits."
  Did Susan make a list of chores? (yes)
  Does Bob need to walk the dog around 2:00 p.m.? (no)
"The dishes in the sink <u>need rinsed</u> and then could you load the dishwasher?" she asked.
Feel free to use the dishwasher if you need to, but only run it once a day."
She continued, "The carpets upstairs <u>need vacuumed</u> but don't worry about the floors down here.
There's always dog hair everywhere."
Bob nodded, "Lemme grab a pen and jot this down."
  Is Bob supposed to do anything in the kitchen? (yes)
  Is Bob supposed to vacuum the hardwood floors? (no)
Susan went on, "I did a load of laundry, but the sheets still <u>need washed</u> with hot water."
Bob interrupted, "Do you need me to, like, use bleach or anything?"
"No, just detergent--measure it so you don't use too much."
  Did Susan ask Bob to wash the bedding? (yes)
  Should Bob use bleach for the laundry? (no)
Susan started on the outside chores.
"The grass <u>needs mowed</u> when it's not too wet.
There should be plenty of gas in the mower.
But there's gonna be sticks on the ground that <u>need picked up</u> before you mow.
Oh, and could you check the flower pots?
They <u>need watered</u> when it gets dry, maybe even this afternoon."
Bob interrupted again, "Is the garden hose hooked up?"

Was grass tall enough to mow? (yes)
Does Bob plan to use the hose for the flower pots? (yes)
Susan nodded and returned to her list. “There are a few more small things.
Bring the mail in every day, and these letters need taken to the post office.
Take the trash out Wednesday night, the truck comes by Thursday morning.”
Susan looked at her watch and sighed “I’m outta here.”
Bob replied “No problem.
Just call me if you think of something else that needs done whileI’m here.”

Should Bob take the trash out Thursday night? (no)

Did Bob complain about all the chores? (no)

Susan and her sister Jill sat in the airport coffee shop, waiting for their flight to be called.
“How’s the house-hunting going?” Susan asked.
“Brutal. I don’t know if I need to find a new agent,” Jill responded.
“The guy we have now hasn’t found us much.”
Was Susan and Jill’s flight cancelled? (no)
Is Jill Susan’s sister? (yes)
“Has he found you anything you’re considering?” Susan asked.
“Just one,” Jill replied.
“I wasn’t blown away, but it has potential, and there hasn’t been anything else, so why not?”
“Why don’t you like it? Is it too small?”
“Oh no, it’s huge. Maybe too big.
It would need scrubbed top to bottom and it’s all very dicey.”
Is Jill considering several houses? (no)
Is the house too small for Jill’s tastes? (no)
“Is it too expensive?” Susan asked next.
“No, it wouldn’t max out our budget.”
“So tell me!” Susan ordered. “Don’t make me guess until I get it right.”
Jill laughed in return. “OK. Well, the house is a mess.
A lot of things will need fixed if we get it.”
“Like what?”
Is Jill worried about the price of the house? (no)
Would Jill have to have repair the house if she buys it? (yes)
Jill sighed, “Well, it needs repainted because all the rooms are in neon colors.
It’s ridiculous.”
Susan laughed, trying to imagine what the house must look like.
“And the floors! Shag carpeting in all the rooms, including the bathroom.
The bathroom carpet would need replaced with tile right away”
Does Jill think the house looks ridiculous? (yes)
Does the carpeting extend to all the rooms of the house? (yes)
Susan smiled, “The homeowners must have… eclectic tastes.”
“That’s exactly what the agent said!
That’s just the part I saw though, half of the house Icouldn’t see.”
“Why not?”

“The homeowners are practically hoarders” Jill confided.

“The upstairs and basement still need emptied which may uncover other issues.”

Has Jill seen the main floor of the house? (yes)

Has the house recently been in a fire? (no)

Susan looked doubtful, “Sounds… questionable. Are you gonna make an offer?”

“What if I can’t find anything else?”

“Maybe something better will turn up soon.”

Has Jill put in an offer for the house? (no)

Is Susan encouraging Jill to buy the house? (no)
