# Supplementary material for: What do we learn when we adapt to reading regional constructions?
Source: PLoS One. 2023 Apr 7;18(4):e0282850. doi: 10.1371/journal.pone.0282850 (PMC10081778; doi:10.1371/journal.pone.0282850)
Supplement: S2 Appendix — (PDF) [file pone.0282850.s002.pdf]

Appendix B. Experiments 2 & 4 Reading Time Stimuli

Each story was preceded by a title, in all caps, as shown. Sentences were presented one word at a time, but comprehension questions appeared all at once; the correct answer for each question is indicated in parentheses.

Phase 1. Words in boldface are double modal tokens (before the slash) or a standard alternative (after the slash). These were presented in normal case to participants.

DAISY’S CAR GOES CLUNK

Daisy’s car made a clunking sound every time she turned left.
Her car had been this way for a week, even after she had taken it in for a tuneup.
She decided to take it back to the auto shop, to get the car looked at again.
Hank, the mechanic who had worked on it previously, came to greet her.

Is this the first time Daisy has brought her car into the auto shop? (no)
Does Daisy’s car make a clunking sound every time she turns left? (yes)

“Hello, ma’am. What brings you back again?” Hank asked.
“The car's drivin' ok,” Daisy said, “but I'm worried about the clunkin' it's doin'.
I was thinkin' you **might could/might just** look at it quick, since it was just here.“
“Well,” Hank said, “we oughtta check it out.
Ya never know. We **might could’ve/ maybe could've** overlooked something.”

Is the mechanic’s name Hank? (yes)
Did the mechanic agree to look over the car again? (yes)

Daisy explained,
“I'm goin’ off on vacation in a week and I'm worried about takin' it on the highway.
I was thinkin’ I **might should/probably should** get it checked again.”
Hank nodded.
Before your trip we **might should better/probably had better** go ahead and have a look.
How's Wednesday fer ya?”

Is Daisy leaving for her vacation in two weeks? (no)
Does the mechanic suggest that he can look at the car on Wednesday? (yes)

Daisy checked her planner: Wednesday was pretty full, but she wanted to cooperate.
“If that's good, I **might could/maybe could** leave it, if someone could run me up to school.
“Hank replied, “If Wednesday's not good, how about Thursday?”
“Thursday it is,” Daisy said.

Q: Did Daisy check her planner? (yes)
Q: Did Hank suggest that Daisy should bring her car in on Friday? (no)

Hank went on:
“I don't think we **might will/probably will** need any more than two hours, tops.”
“On Thursday, could someone still drive me to school? Daisy asked.
“Just bring it and we'll run you up to school,” Hank replied.
“How about pull the hood latch and let's take a look.”
Hank pulled up the hood of the car.
“No, I thought I **might could/maybe could** see what was loose, but I don't see nothin'.

We'll have a closer looksee Thursday.”

Did Daisy agree to have her car looked at on Wednesday? (no)

Did Hank find the cause of the noise by looking under the hood? (no)

Daisy hesitated and asked,

“**May could/Perhaps could** you give me a discount since I brought it in before?”

Hank replied, “Well, I **might had better/really had better** ask my boss.“

“Yes, please,” said Daisy.

“I’m sorry for the inconvenience, ma’am.

But you know, I **may can/could probably** do an oil change for free,” said Hank.

“Thanks!,” said Daisy, “I’ll see you Thursday.”

Did Hank offer a free oil change to Daisy? (yes)

<u>Phase 2. </u>The infelicitous double modals precede the slash and the regionally acceptable double modals are presented after the slash. The manipulated words are in boldface here, but were in normal font for the experiment.

THE CANOE TRIP

Belle knocked on Annabeth’s door.

She was in rush, but still wanted to stop by and see her friends.

Annabeth and Janie had been planning a canoe trip.

Annabeth opened the door.

Is Janie going on a canoe trip? (yes)

Is Carl going on the canoe trip? (no)

“Hey Belle! Are ya ready?”

“Come on in, you **can might/might can** help us pack.“

‘I’m actually kinda busy right now,” said Belle.

“I wanna help, but I don’t wanna be late for work.

Hey I think I **could might/might could** make sack lunches for y'all later.”

“That’d be great,” replied Annabeth and Janie at once.

Would Belle make sack lunches later? (yes)

Are the friends going out to eat at a restaurant after the canoe trip? (no)

Janie added, “Belle, you **could might/might could** help me load the canoe onto the car.”

‘I’m so sorry, I don’t have time,” Belle replied. “But what do you wanna eat on the trip?

Oh ya know, y’all **should might/might should** tell me your allergies.”

“No peanuts,” said Annabeth. “Janie’s allergic.”

“How about turkey sandwiches?”

“Sounds good,” replied Annabeth.

“And I guess I **should may/may should** bring napkins too,” said Belle.

“Good thinkin’,” smiled Annabeth.

Did Janie ask Belle to help put the canoe on the car? (yes)

Is Janie allergic to milk? (no)
