# Supplementary material for: What do we learn when we adapt to reading regional constructions?
Source: PLoS One. 2023 Apr 7;18(4):e0282850. doi: 10.1371/journal.pone.0282850 (PMC10081778; doi:10.1371/journal.pone.0282850)
Supplement: S3 Appendix — (PDF) [file pone.0282850.s003.pdf]

# Appendix C. Acceptability Judgment Stimuli from Experiments 3 & 4

For the critical items below, there were 3 versions of each sentence: one that was acceptable in Standard American English (a), one that was acceptable in the regional dialect (b), and one that was ungrammatical in either (c).

<u>*Needs* Construction</u>

(1) a. The bike tires need to be inflated so we can go on a ride.
b. The bike tires need inflated so we can go on a ride.
c. The bike tires need inflate so we can go on a ride.

(2) a. The computer needs to be rebuilt after you take it apart.
b. The computer needs rebuilt after you take it apart.
c. The computer needs rebuild after you take it apart.

(3) a. The cat's claws need to be clipped because they are too long.
b. The cat's claws need clipped because they are too long.
c. The cat's claws need clip because they are too long.

(4) a. The computer program needs to be debugged before I hand it in.
b. The computer program needs debugged before I hand it in.
c. The computer program needs debug before I hand it in.

(5) a. Head wounds need to be treated as soon as possible.
b. Head wounds need treated as soon as possible.
c. Head wounds need treat as soon as possible.

(6) a. The turkey needs to be carved before it can be served.
b. The turkey needs carved before it can be served.
c. The turkey needs carve before it can be served.

(7) a. Small potatoes need to be boiled before they can be used in the soup.
b. Small potatoes need boiled before they can be used in the soup.
c. Small potatoes need boil before they can be used in the soup.

(8) a. The news articles need to be edited before they are published.
b. The news articles need edited before they are published.
c. The news articles need edit before they are published.

(9) a. The screws need to be tightened after they are all in place.
b. The screws need tightened after they are all in place.
c. The screws need tighten after they are all in place.

(10) a. His beer glass needs to be filled because it's empty again.
b. His beer glass needs filled because it's empty again.
c. His beer glass needs fill because it's empty again.

(11) a. These bills need to be paid before the end of the month.
b. These bills need paid before the end of the month.
c. These bills need pay before the end of the month.

(12) a. The piano needs to be tuned so the musicians can play.
b. The piano needs tuned so the musicians can play.
c. The piano needs tune so the musicians can play.

(13) a. The fire needs to be stirred to keep it from burning out.
b. The fire needs stirred to keep it from burning out.
c. The fire needs stir to keep it from burning out.

(14) a. The patio needs to be decorated for the party.
b. The patio needs decorated for the party.
c. The patio needs decorate for the party.

(15) a. The film needs to be approved before it can be released.
b. The film needs approved before it can be released.
c. The film needs approve before it can be released.

(16) a. The shirt needs to be mended so I can wear it tonight.
b. The shirt needs mended so I can wear it tonight.
c. The shirt needs mend so I can wear it tonight.

(17) a. The lock needs to be checked so we can be sure the apartment is secure.
b. The lock needs checked so we can be sure the apartment is secure.
c. The lock needs check so we can be sure the apartment is secure.

(18) a. The term paper needs to be revised before tomorrow morning.
b. The term paper needs revised before tomorrow morning.
c. The term paper needs revise before tomorrow morning.

<u>Double modal construction</u>

(19) a. You should eat before you go to work.
b. You might should eat before you go to work.
c. You should might eat before you go to work.

(20) a. I can ask my boss for the day off on Friday.
b. I might can ask my boss for the day off on Friday.
c. I can might ask my boss for the day off on Friday.

(21) a. Well, John could pick some up from the store if you really need them.
b. Well, John might could pick some up from the store if you really need them.
c. Well, John could might pick some up from the store if you really need them.

(22) a. Since Steve can't, I could give Sarah a ride home.
b. Since Steve can't, I might could give Sarah a ride home.
c. Since Steve can't, I could might give Sarah a ride home.

(23) a. We might go up there next Saturday.
b. We might can go up there next Saturday.
c. We can might go up there next Saturday.

(24) a. Let's at least talk about what could be made better about the private sector.
b. Let's at least talk about what might could be made better about the private sector.
c. Let's at least talk about what could might be made better about the private sector.

(25) a. If it rains, you might wish you had that umbrella with you.
b. If it rains, you might would wish you had that umbrella with you.
c. If it rains, you would might wish you had that umbrella with you.

(26) a. I should turn this thing over to Ann.
b. I might should turn this thing over to Ann.
c. I should might turn this thing over to Ann.

(27) a. Julie said she thinks she may come tonight, if she can find something to wear.
b. Julie said she thinks she may can come tonight, if she can find something to wear.
c. Julie said she thinks she can may come tonight, if she can find something to wear.

(28) a. If you're really worried about a non-alcoholic option, you could make some sweet tea.
b. If you're really worried about a non-alcoholic option, you might could make some sweet tea.
c. If you're really worried about a non-alcoholic option, you could might make some sweet tea.

(29) a. I think I have some grants you could apply for.
b. I think I have some grants you might could apply for.
c. I think I have some grants you could might apply for.

(30) a. If he wants to make his daughter's birthday party, Jim should be home from work by 4:30.
b. If he wants to make his daughter's birthday party, Jim might should be home from work by 4:30.
c. If he wants to make his daughter's birthday party, Jim should might be home from work by 4:30.

(31) a. Most people can't tell if Daddy is kidding them or not, but Bill could tell you if he's serious.
b. Most people can't tell if Daddy is kidding them or not, but Bill might could tell you if he's serious.
c. Most people can't tell if Daddy is kidding them or not, but Bill could might tell you if he's serious.

(32) a. I reckon I should try to get a little bit more sleep.
b. I reckon I might should better try to get a little bit more sleep.
c. I reckon I should might better try to get a little bit more sleep.

(33) a. When she was three, Rebecca often wondered if she could write on the walls.
b. When she was three, Rebecca often wondered if she might could write on the walls.
c. When she was three, Rebecca often wondered if she could might write on the walls.

(34) a. There could be water in that old well.
b. There might could be water in that old well.
c. There could might be water in that old well.

(35) a. I asked him if he would have it ready by one o'clock.
b. I asked him if he might would have it ready by one o'clock.
c. I asked him if he would might have it ready by one o'clock.

(36) a. If I were you, I would try digging over by that creek.
b. If I were you, I might would try digging over by that creek.
c. If I were you, I would might try digging over by that creek.

Filler sentences

All of the filler sentences contained other regionally acceptable constructions (i.e., not the *needs* construction or double modals). These sentences were based on examples from the Yale Grammatical Diversity Project (https://ygdp.yale.edu/).

(1) Jane will try and kill mosquitoes.

(2) I try and finish an assignment every day.

(3) Alls Alice brought to the party was bread.

(4) Alls we want to do is sing a song.

(5) We got a lot of problems right now.

(6) They are leaving on a great adventure; you should go with.

(7) Here's you a piece of pizza.

(8) Here's John a glass of iced tea.

(9) By the time I am done dinner, I don’t want my side snack.

(10) This will be particularly important once you’re done the tattoo and need to leave the shop.

(11) Of course, it’s possible they could have been a mill there.

(12) They’s about six or seven guitar players here.

(13) I done finished that already.

(14) I can’t believe that dance class is canceled after I done wanted to take it for five years.

(15) He was looking to buy him a house for his family.

(16) Mary would love her some flowers.

(17) He has plenty of free time, so he exercises a lot anymore.

(18) Gas is pretty expensive anymore.
