# Supplementary material for: What do we learn when we adapt to reading regional constructions?
Source: PLoS One. 2023 Apr 7;18(4):e0282850. doi: 10.1371/journal.pone.0282850 (PMC10081778; doi:10.1371/journal.pone.0282850)
Supplement: S4 Appendix — (PDF) [file pone.0282850.s004.pdf]

Appendix D. Construction familiarity responses

After reading the two stories, participants were asked two questions about their familiarity with the needs construction: how often had they heard it (never; only once or twice; regularly, but not often; frequently; very often), and in how many different contexts they had experienced it (never heard it; only in books/movies/TV; used by someone they do not really know; one or more of their friends uses it; one or more of their relatives uses it; use it themselves). This measure provides a rough index of prior exposure to the construction in our samples.

It is important to note that these responses were collected after completing the primary experiment and may have been influenced by exposure during the experiment.

The two types of familiarity responses (heard familiarity and context familiarity) are summarized in separate histograms for each experiment. The data reported are the number of participants who selected each labelled option, irrespective of participant group.

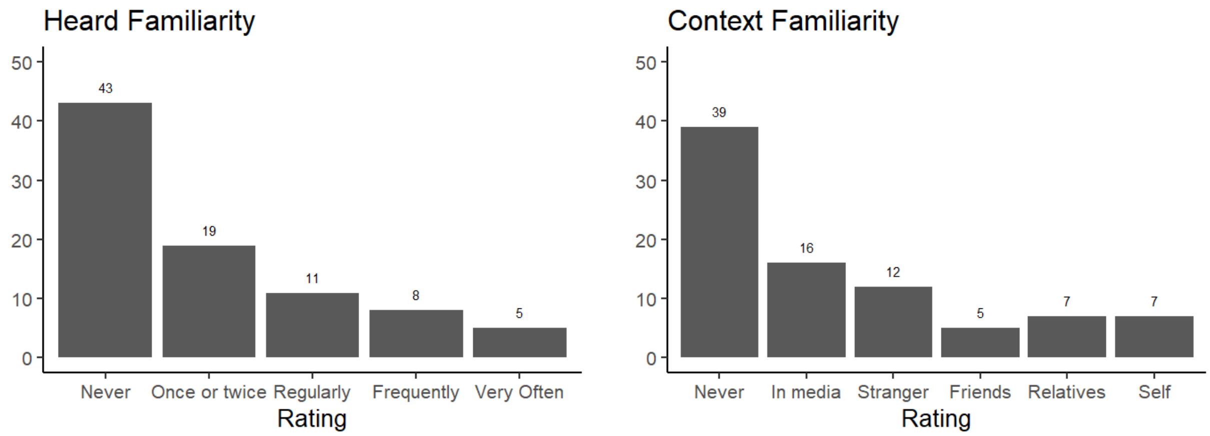

Fig D1. Experiment 1 participants' prior familiarity with the *needs* construction.

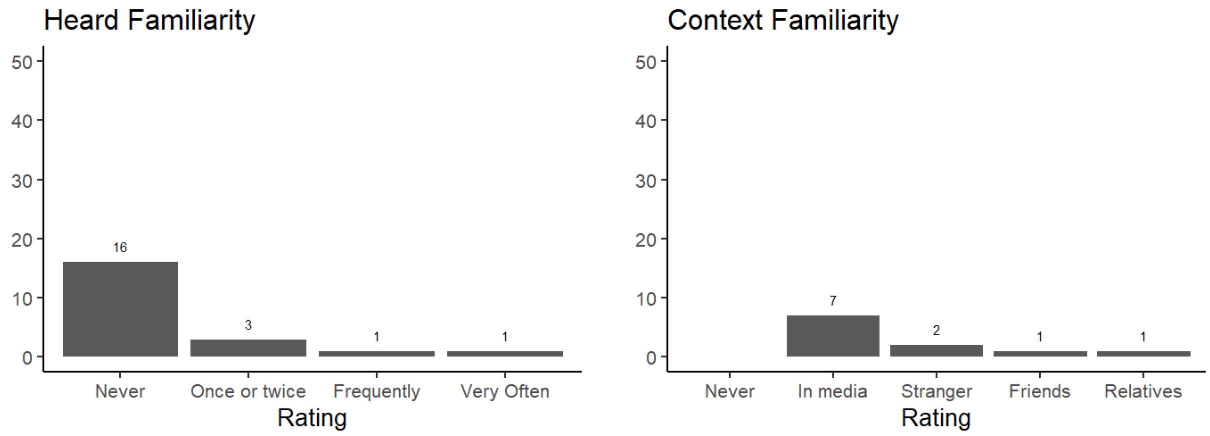

Fig D2. Experiment 2 participants' prior familiarity with the double modal construction. A procedural problem lead to a large amount of missing data; complete data is only available from 17 participants, partial data from 56 participants, and no data from the remaining 56 participants.

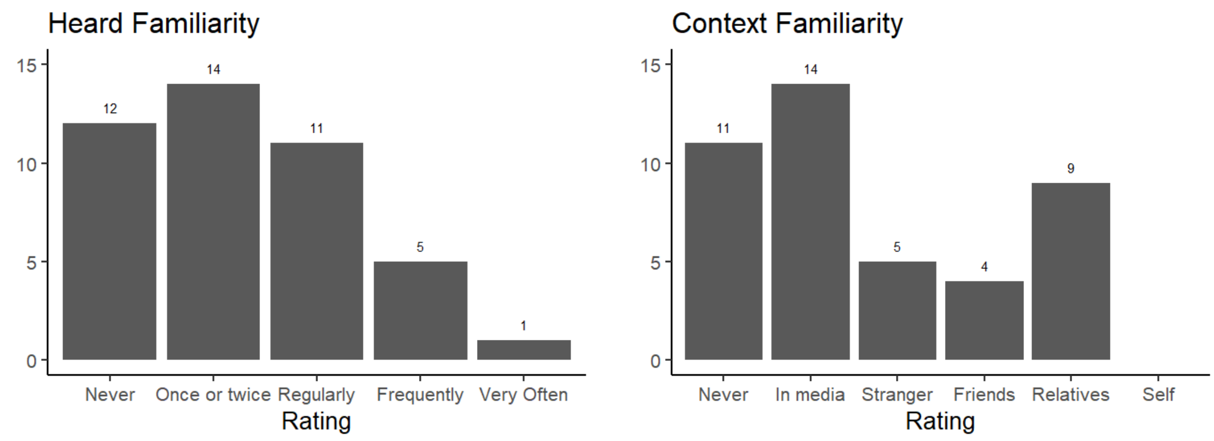

Fig D3. Experiment 3 participants' familiarity with the *needs* construction.

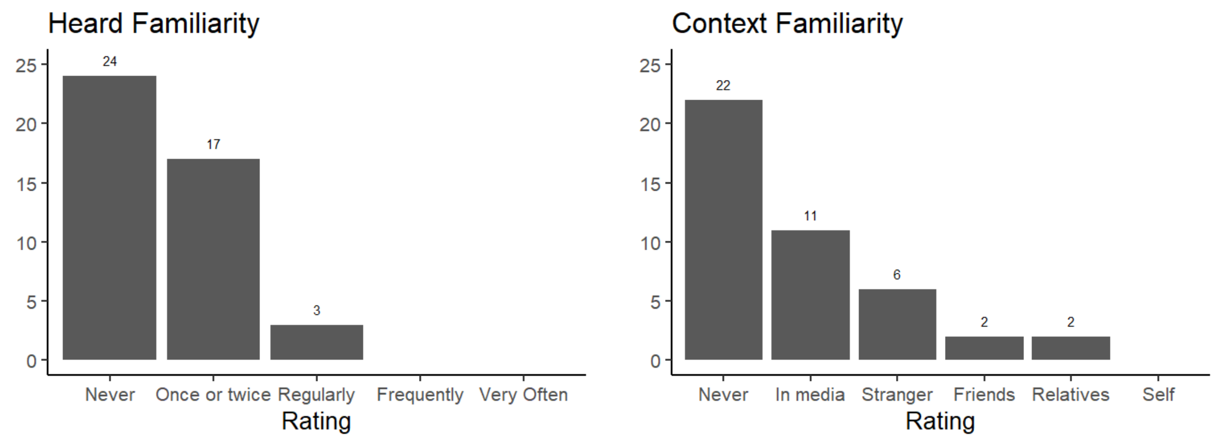

Fig D4. Experiment 4 participants' familiarity with the double modal construction.
